# Linking retinal sampling in neural encoding models to temporal profiles of visual processing in humans

**DOI:** 10.1371/journal.pcbi.1014371

**Published:** 2026-06-30

**Authors:** Niklas Müller, Hongye Chen, Sofie Wahlberg, H. Steven Scholte, Iris I. A. Groen

**Affiliations:** 1 Department of Psychology, University of Amsterdam, Amsterdam, The Netherlands; 2 Informatics Institute, University of Amsterdam, Amsterdam, The Netherlands; Utrecht University: Universiteit Utrecht, NETHERLANDS, KINGDOM OF THE

## Abstract

Retinotopic tuning of neural populations is a key organizing principle of human visual cortex. However, state-of-the-art models that predict neural recordings based on task-optimized Convolutional Neural Networks (CNNs) do not take this retinotopic organization into account. Furthermore, while retinotopic tuning in visual cortex has been studied extensively using functional magnetic resonance imaging, the temporal dynamics of processing information from distinct parts of the visual field are less well understood. Here, we reveal distinct temporal profiles for foveal and peripheral visual information processing by implementing multiple spatial sampling strategies on feature maps of CNNs into encoding models that predict human electroencephalography (EEG) responses. Using large, high-quality natural scene images, we show that processing of peripheral information precedes that of foveally sampled information. This temporal difference is best modeled when applying a differential spatial transform to CNN feature maps that is derived from empirical measurements of human retinal ganglion cells. We directly confirm this temporal difference experimentally by mutually exclusive stimulation of foveal and peripheral visual field regions. Last, we introduce a novel, data-driven method of recovering visual field information from neural data, highlighting and quantifying spatial, retinotopic information contained in temporally specific EEG recordings. Together, these results provide novel neural evidence for a temporal coarse-to-fine visual processing hierarchy in the processing of natural images that is directly linked to distinct spatial information sampling. Aligning the spatial sampling of humans and CNN encoding models not only improves predictions of neural responses but also demonstrates that EEG recordings contain a significant amount of temporally encoded retinotopic information. We make our large-scale EEG dataset including high-resolution natural scene images publicly available to enable future research into naturalistic visual processing.

## Author summary

The human brain processes information about the physical environment. This information is captured by several sensory organs which, through their structure, determine the format of the input to the brain. In human visual perception, this is the structure of the retina such as the number of photoreceptors or ganglion cells. Their densities differ across the retina with many more receptors and cells in the fovea (i.e., the point of gaze fixation) compared to the periphery. As such, the brain receives more information coming from the fovea compared to peripheral regions. Here, we identify differences between foveal and peripheral processing and reveal how this unfolds over neural processing time using electroencephalography recordings during natural scene viewing. Combined with state-of-the-art modeling techniques that predict brain activity with deep neural networks, we show that the brain is processing peripheral visual information before foveal information. Crucially, this temporal shift was only detected when stimuli were presented large-field, thereby better approximating the percept of the real, natural world. These findings suggest that the brain processes information about the environment in stages, forming a hierarchy underlying visual perception.

## 1 Introduction

A large part of human visual cortex is retinotopically organized [1–5]: neurons in visual cortex preferably respond to visual stimulation at a specific location in the visual field (their Receptive Field, RF), and neurons are arranged on the cortical sheet such that nearby neurons have RFs at nearby locations. As a consequence, population responses recorded using functional magnetic resonance imaging (fMRI) [6] or electrophysiology [7] are well predicted by forward models that simulate spatially local sampling of visual information by means of a population Receptive Field (pRF) of a certain location and size. Such pRF models successfully retrieve well-known properties of retinotopic maps, such as increasing receptive field sizes with visual eccentricity [8]. However, while pRF models accurately predict brain responses to simple, spatially varying stimuli, they perform less well for complex, full-field natural scenes [9], presumably because they lack tuning to specific visual features within the pRF location [10].

In contrast, encoding models built on feature maps of Convolutional Neural Networks (CNNs) [11] predict responses to natural scenes with high accuracy across multiple species and modalities, including monkey electrophysiology [12,13], human MEG [14], BOLD [15,16], and EEG [17]. However, these models lack the crucial inductive bias of retinotopic tuning that make pRF models such powerful prediction models. CNNs perform spatially local computations by means of convolutional kernels, but these kernels are uniformly applied across the input image. This uniform sampling originates from the weight sharing of convolutional features [18], which was a key milestone in advancing computer vision models, enabling them to learn and perform tasks such as spatially invariant object recognition and scene segmentation [19–21]. However, it prohibits differential sampling of visual information across the visual field by learning features at individual spatial locations, which is a hallmark property of biological vision [22]. Thus, while CNNs have been successful in learning human-like tasks, their network architecture, specifically how they spatially sample the visual field, differs significantly from that of the human visual cortex.

Here, we investigate whether combining spatially local sampling (inspired by pRF models) with rich feature representations (from CNNs) results in better predictions of neural responses to full natural image stimuli. Previous work has demonstrated how this combination can be used as an alternative approach for pRF mapping in human fMRI recordings [10]. Whether encoding models of other brain imaging modalities also benefit from integrating spatially local sampling, remains unclear. Moreover, unlike fMRI, temporally-resolved recordings such as EEG can reveal temporal dynamics of visual processing [23]. Here, we focus on one key form of differential spatial sampling in human visual processing facilitated by retinotopy: foveal versus peripheral processing.

Differential foveal and peripheral processing in human vision originates from the retinogeniculate parvocellular and the magnocellular pathways [25], which process inputs with high-acuity from the central visual field and low-acuity from the peripheral visual field, respectively [26]. A higher density of receptors in the parvocellular pathway is matched by assigning substantially more brain volume to the processing of foveal information (cortical magnification) [27–29]. However, the magnocellular pathway operates at a faster timescale than the parvocellular pathway, evident in higher temporal flicker sensitivities in peripheral vs. foveal processing, for example [30–32].

Although less represented in cortex, peripheral information is thought to serve important roles in scene perception and spatial navigation [33–37]. For example, the fast magnocellular pathway [38] is thought to aid rapid recognition of scene gist [39–41] and motion detection [42]. A temporal difference between peripheral and foveal processing may facilitate coarse-to-fine (CTF) hierarchies in visual processing, by allowing peripheral (’coarse’) computations to precede foveal (’fine’) computations. However, there are many other putative neural mechanisms for CTF processing than spatial sampling, ranging from spike-timing based codes to Bayesian sequential decision making [43]. Moreover, most prior evidence for CTF processing, despite being convergent across studies, largely originates from behavioral experiments or experiments performed on artificial stimuli [43–47], rather than natural scenes. Since neural responses can differ under stimulation with artificial vs. natural stimuli [48–52], it remains important to establish a periphery-to-fovea processing hierarchy under more naturalistic settings. Here, we use encoding modeling to identify temporal signatures of neural processing of peripheral and foveal scene parts using natural visual stimuli.

First, we show that incorporating spatially local sampling into a CNN-based encoding model can identify temporal signatures of foveal and peripheral processing in human EEG responses while participants view natural, full-field scenes. To test whether the retinotopic organization of early visual cortex influences EEG signals, we separated foveal from peripheral information in CNN feature maps and tested whether these mutually exclusive inputs to the encoding model yield different predictions of EEG signals to full images better across time points and electrodes. We find clear temporal differences when using distinct spatial features, where peripheral information explains early time points better and foveal information explains later time points better. Second, we show that spatially reweighting CNN activation maps based on empirically derived densities of retinal ganglion cells (RGCs) holistically improves EEG encoding performance at both early and late time points. Critically, these results are robust across multiple CNN architectures and multiple CNN training datasets.

Next, to experimentally validate these modeling results, we collected additional EEG recordings: we selectively stimulated mutually exclusive, central and peripheral areas of the visual field and show that we can robustly replicate the temporally distinct encoding performances associated with the processing of central and peripheral information. Finally, we introduce a novel, fully data-driven method to identify and quantify how each spatial location in the visual field contributes to the overall neural dynamics elicited by high-resolution natural images.

In sum, by comparing CNN-based encoding models with different spatial sampling strategies, we find that information from distinct visual field regions elicits temporally distinct neural signatures. These findings support a global-to-local processing hierarchy, consistent with a coarse-to-fine theory of visual perception. Importantly, we demonstrate that using a retina-inspired spatial transform on the encoding model inputs unifies these temporally specific dynamics and thereby significantly improves neural encoding performance of EEG responses relative to standard, non-spatially weighted encoding models.

## 2 Results

### 2.1 Modeling differential spatial sampling reveals temporal profile of processing natural scenes

**Fig 1:**
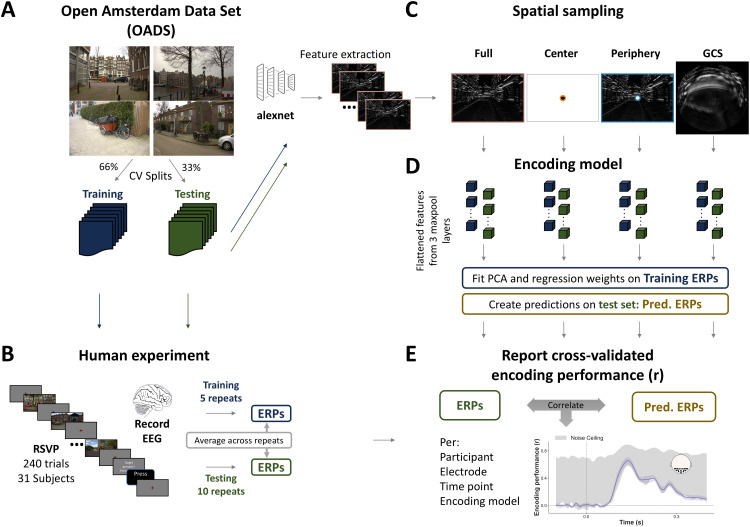
Encoding models for predicting human EEG responses to natural images. **A** 4680 high resolution, natural outdoor scene images from the Open Amsterdam Data Set were divided into two non-overlapping sets: a training (66%) and a test set (33%). **B** We recorded EEG data during a rapid serial visual presentation (RSVP) experiment with an indoor-scene detection task. **C** Four forms of spatial sampling were applied post-hoc to the extracted feature maps from 3 layers of a task-optimized AlexNet for each stimulus image: Full (baseline), Center (keeping the central 0.5%), Periphery (removing the central 0.5%, and GCS (applying the retinal ganglion cell sampling [24] transformation). **D** Principal Component Analysis (PCA) was applied to flattened versions of each of the four feature maps, after which we fitted a linear regression model for each subject, electrode, and time point separately to predict the measured event-related potential (ERP) amplitudes to the training set images. We then applied the fitted regression weights to the held-out features from the test set to generate new ERP amplitude predictions. **E** We compared the test set predictions from each of the four models to the recorded ERP amplitudes using Pearson correlation (r). We report this cross-validated encoding performance and perform statistical difference tests across subjects to determine the highest-performing encoding model per time point and electrode.

We first investigated whether selectively sampling from different parts of CNN feature maps improves encoding model predictions of human EEG response amplitudes to high-resolution, large-field natural scene images (Fig 1A), shown in a rapid serial visual presentation (RSVP) experiment (Fig 1B). We extracted feature maps from 3 layers of a task-optimized AlexNet for each stimulus image and subsequently created four different versions for the same set of extracted features (Fig 1C): a baseline condition using the full feature map (“Full”); keeping only the central 0.5% of the feature map while discarding the rest (“Center”); removing the central 0.5% of the feature map while keeping the rest (“Periphery”); and applying a retinal ganglion cell sampling (GCS) [24] transformation to the full feature map (“GCS”). For each type of feature selection (Full, Center, Periphery, GCS), we fit a separate encoding model to predict single-image EEG responses to the training images (Fig 1D). We then assessed to what extent encoding performance changed for differential sampling models (Center, Periphery, GCS) compared to the baseline model (Full) for each participant, time point and EEG electrode (Fig 1E).

#### 2.1.1 Spatial feature selection affects EEG encoding performance.

**Fig 2:**
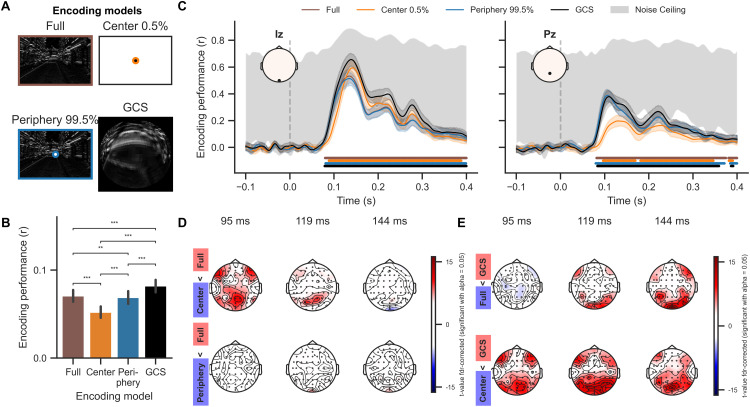
Spatial feature selection improves encoding model predictions. **A** Examples of spatial transformations of CNN feature maps. Transforms either keep the full feature map: “Full”; take a center-crop: “Center”; take a periphery-crop: “Periphery,” i.e., removing exactly the center-crop; or apply a retinal ganglion cell sampling (GCS) magnifying the center while reducing, yet maintaining peripheral information: “GCS”; for details see Methods section 4.2.1. **B** Cross-validated encoding performance (Pearson’s correlation between predicted and real ERP amplitudes) averaged across all time point and all electrodes per participant per encoding model. Bars show average (with error bar indicating 95% confidence intervals) across subjects. Asterisks indicate statistically significant differences of Wilcoxon signed-rank tests across subjects between all pairs of encoding models. **C** Average cross-validated encoding performance across subjects over time for electrodes Iz (left) and Pz (right) for all four encoding models. Shaded gray areas show the estimated lower and upper bound of the noise ceilings per electrode, per time point, averaged across subjects. Asterisks indicate statistically significant encoding performance per encoding model (permutation t-test). Insets indicate the electrode’s location on the scalp. **D-E** Topoplots show the FDR-corrected t-values of a one-sample t-test testing for statistical difference from 0 of the difference of correlations between two conditions. Tested pairs of conditions are indicated on the left of the topoplots (colors indicating the direction of the statistical outcome), for time points 95 ms (first column), 119 ms (second column), and 144 ms (third column).

Consistent with our hypothesis, the GCS model (Fig 2A) outperforms all three other models on the overall encoding performance averaged across all participants, electrodes and time-points (Fig 2B: Wilcoxon signed-rank test for GCS against Full [W = 21, p = 4.163e-7], Center [W = 1, p = 1.863e-9], Periphery [W = 21, p = 4.163e-7]). Interestingly, while both the Full model (W = 29, p = 1.620e-6) and the Periphery model (W = 37, p = 5.318e-6) outperform the Center model, the Center model still performs remarkably well, given that it only receives 0.5% of the information that the Full model receives. At the same time, removing the center in the Periphery model slightly reduces, but does not completely eradicate, encoding performance, suggesting that peripheral information has explanatory power independent of the center. The superior performance of the GCS model relative to all three other models suggests it more accurately approximates how foveal and peripheral information are reflected in the EEG signal.

To better understand the spatiotemporal dynamics underlying these differences in encoding performance, we illustrate below the individual contributions of foveal and peripheral processing to the EEG signal by comparing encoding performances across the full time course for selected electrodes Iz and Pz (representing the most posterior and a more central electrode; see line plots in Fig 2C), as well as across the full scalp for selected time points 95 ms, 119 ms, 144 ms (roughly representing an early, intermediate, and late time point of feedforward processing, see topoplots in Fig 2D and 2E). We first describe the relative differences in encoding performance for the Center/Periphery models versus the Full baseline model, which helps contextualize the overall superior performance of the GCS model.

#### 2.1.2 Foveal information primarily drives occipital electrodes.

At occipital electrode Iz, all four models exhibit significant encoding performance starting around 80 ms after stimulus onset (Fig 2C, left). Encoding performance for the Center model is higher than both the Full model and the Periphery model starting at approximately 120 ms. Additionally, the performance of the Periphery model is lower than the Full model at time points between around 120 and 145 ms. This suggests that ERP responses at Iz are predominantly driven by foveal information.

A qualitatively different pattern is observed at peri-occipital and central electrodes. Performances of the Full and Periphery model are significant at time points later than 80 ms for Pz (Fig 2C, right), while the Center model is only significant after 95 ms at Pz. For central electrode Pz, performance of the Periphery and Full model are higher than the Center model for time points earlier than approximately 145 ms. This suggests that full-field information contributes more to the ERP response variance in these electrodes, early in time. Results for the average encoding performance across groups of electrodes (all occipital, all central) show a similar pattern and are included in S11 Fig.

To test which spatial selection model has the highest encoding performance at distinct moments in visual processing, we performed pairwise t-tests across participants at every electrode for three time points (95, 119, and 144 ms after stimulus onset). We found that the Full model significantly outperforms the Center model at central electrodes at 95 ms (see Fig 2D, top). This pattern disappears at 119 ms, when both models perform similarly across electrodes. At 144 ms, the Center model in turn outperforms the Full model at occipital electrodes highlighting again that ERP responses at these sites encode central information much more than peripheral information. Overall, these results show that at early time points, the best encoding model uses information from the full visual field, whereas at later time points usage of exclusively central information is beneficial.

#### 2.1.3 Early processing of peripheral information.

Next, to test if there are any time points or electrodes that show an advantage of processing peripheral information (rather than full-field information), we compare the encoding performances of the Full model and the Periphery model (see Fig 2D, bottom). At early time points (e.g., 95 ms) there are no electrodes for which there is a significant difference between the Full model and the Periphery model. Nevertheless, at both later time points (119 and 144 ms) the Full model outperforms the Periphery model at the most occipital electrodes. Notably, the Periphery model uses feature maps where only 0.5% of information in the center were removed. This removal yields significantly worse predictions compared to using the full feature map for the most posterior electrodes. Thus, even with 99.5% of shared information, there is a discrepancy between the encoding model predictions of the Full and the Periphery model.

Comparing the Center model against the Full model, as well as the Periphery model against the Full model, allows us to assess the impact of removing either central information or peripheral information. Overall, we observe that peripheral information is sufficient and necessary to explain the majority of variance at early time points (95 ms). In turn, using peripheral information leads to reduced encoding performance at later time points while central information is now sufficient and necessary for good model performance.

#### 2.1.4 Non-uniform retinal sampling holistically predicts EEG signals.

So far, we have seen that responses at specific electrodes and time points are largely driven by either central or peripheral information. Further, we see that, on average, the GCS model that differentially combines central and peripheral information outperforms all other models. This raises the question whether the GCS model can account for the electrode and time point specific responses better than the other models.

We find that the GCS model significantly outperforms the Full model at time points later than 95 ms at occipital and temporal electrodes (see Fig 2E). Interestingly, the GCS model also outperforms the Center model at the later time points (119 and 144 ms), which, as shown above, was significantly better than the Full model at these time points. Only at the earliest time point (95 ms), the Full model still slightly outperforms the GCS model at central electrodes, suggesting it does not perfectly capture differential foveal and peripheral contributions to the EEG response. The GCS transform uses a magnification factor that we fixed to 20 degrees for this work, as was originally suggested [24] based on empirical measurement. However, varying this factor shows that higher encoding performance can be achieved also at early time points when using a smaller magnification factor, in line with a stronger representation of peripheral information. Results for varying the magnification factor are included in S6 Fig.

Overall, our results show that spatial reweighting using the GCS transform either outperforms or performs on par with both the Center and the Full model at almost all time points and electrodes, and outperforms the Periphery model at all time points. This demonstrates that the temporal dynamics of natural image processing are best accounted for when foveal and peripheral information are weighted according to the sampling density of the human retina.

To validate that our findings about temporal differences in the encoding of foveal and peripheral information, we repeated our analyses for encoding models based on other ImageNet-trained CNN architectures (ResNet18, ResNet50, ConvNeXt; [21,53]), on CNNs trained on scene categorization on a dataset of scene images (Places365; [54]) instead of object images, as well as on untrained CNNs. Encoding results for these models are shown in S7–S9 Figs. Further, we show that results do not different qualitatively when using oval or rectangular crops with a comparable size, instead of circular crops (see S10 Fig).

#### 2.1.5 Temporally distinct processing of spatially distinct features.

Our results suggest that distinct parts of the visual field have different temporal processing profiles in the EEG signal, with peripheral/full-field information being processed slightly earlier than central information. While the Central and Peripheral encoding models are based on distinct, non-overlapping parts of the CNN feature maps, they may still share a substantial amount of similar, correlated features, potentially underestimating this temporal distinction. To better understand the extent of how temporally distinct the processing of foveal and peripheral information is, we calculated partial correlations for the predictions of the Center, Periphery, GCS, and Full model, respectively, while using the predictions of one of the other models as the covariate (see methods section 4.2). Thus, variance that is explained by both model predictions is excluded from the correlation calculation.

**Fig 3:**
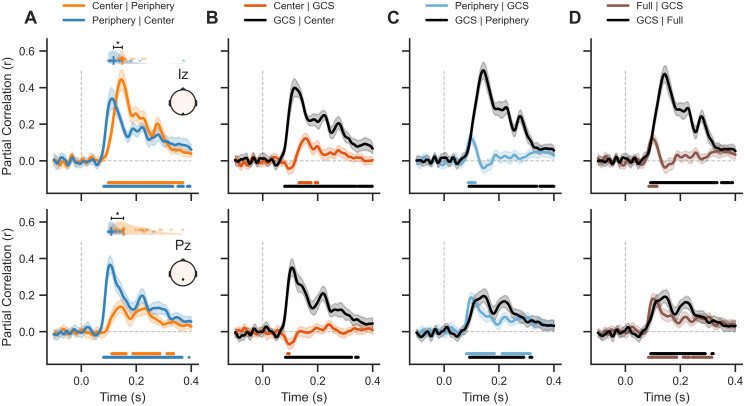
Temporally distinct ERP predictions by spatially distinct sampling, unified by retinal spatial reweighting Plots show partial correlations (r) over time for the following pairs of encoding models: A Center vs Periphery model: peripheral information predict early time points while central information predicts later time points. Asterisks at the bottom indicate significant correlations across subjects per condition (permutation t-test). Asterisks at the top indicate the time point of the maximum correlation per participant, as well as the distribution across participants for the Center condition and Periphery condition with a black asterisk indicating a significant difference between the two conditions (Wilcoxon signed-rank test). **B** Center vs. GCS model: the GCS model captures almost all variance that the Center model explains. **C** Periphery vs. GCS model: the GCS model captures almost all variance that the Periphery model explains, but not vice-versa. **D** Full vs. GCS model: the GCS model captures almost all variance that the Full model explains, but not vice-versa. Bottom asterisks in B-D same as in A.

Partial correlations of Center and Periphery models reveal distinct temporal patterns: the Periphery model explains more unique variance at early time points and less unique variance at time points between ~120 and 145 ms at both Iz and Pz (see Fig 3A). In turn, the Center model explains unique variance delayed in time: the first significant time point is 93 ms for Iz and 102 ms for Pz compared to 76 ms and 75 ms for the Periphery model for Iz and Pz, respectively. To quantify the temporal delay between Center and Periphery, we statistically compare the time point of maximum partial correlation across participants and find that the Periphery model explains most unique variance significantly earlier in time compared to the Center model, for both electrodes. However, the temporal delay of the Center model is most prominent at occipital electrode Iz, while at the more central electrode Pz, the unique variance of the Center model with respect to the predictions of the Periphery model is substantially lower. While these temporal differences where already visible in Fig 2, this analysis more clearly reveals the magnitude of the temporal differences between central and peripheral processing by removing any overlapping explained variance.

#### 2.1.6 GCS captures both central and peripheral processing.

To test whether the GCS model adequately combines the unique contributions of central and peripheral information, we also computed the partial correlation of the GCS model with the Center, Periphery, and Full model, respectively. The GCS model explains unique variance over the Center model at earlier time points as well as later time points at electrode Iz and Pz (see Fig 3B), showing that it captures almost all the variance that the Center model explains - plus variance that the Center model cannot explain, most likely because the GCS model also uses peripheral information. Furthermore, the GCS model explains unique variance over the Periphery model at electrodes Iz and Pz at both early and late time points (see Fig 3C), again highlighting that the GCS model captures almost all variance that is explained by the Periphery model plus additional variance that the Periphery model cannot explain. However, the Periphery model still explains some unique variance at very early time points that the GCS model does not seem to capture. These findings suggest that the spatial reweighting applied by the GCS transform is largely, however not completely adequate to capture peripheral processing. We further elaborate on a potential solution for this in Discussion section 3.4.

Lastly, we tested whether the unique variance explained by the GCS model over the Center and Periphery models, is indeed a contribution of the GCS-transform and is not present in the Full model: thus, we tested the GCS model predictions against the Full model predictions (see Fig 3D) and find that the GCS model explains substantially more unique variance than the Full model at all time points for Iz. For Pz, the Full model explains some unique variance at very early time points, similar to the unique variance explained by the Periphery model. Together, these partial correlation analyses suggest that the processing of central and peripheral information is temporally distinct and that both temporal dynamics can almost fully (with the exception of very early peripheral processing) be captured using retinal spatial reweighting.

### 2.2 Experimental manipulation of visual field stimulation confirms temporally distinct center-periphery processing

Using differential spatial sampling of CNN feature maps, we showed that central and peripheral information contribute uniquely to the explained variance of linearized encoding models predicting human EEG responses during viewing of large-field natural scenes. Importantly, this did not necessitate creating spatially varying stimuli to identify the underlying spatial tuning in EEG signals. This demonstrates the power of encoding models at uncovering the signatures of retinotopic organization in EEG signals without an experimental manipulation of visual stimulation to the participants.

**Fig 4:**
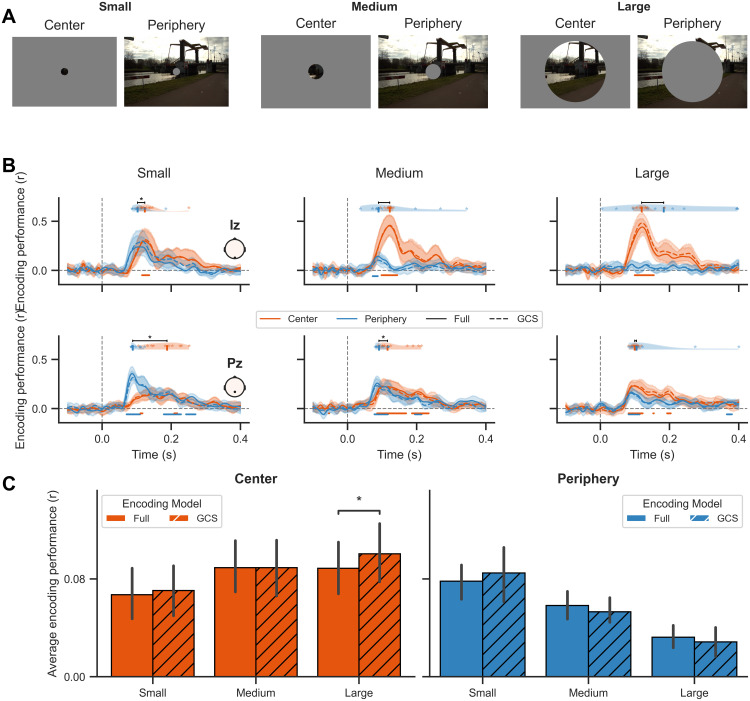
Spatially distinct visual stimulation elicits temporally distinct ERPs. **A** Example stimuli for center and periphery conditions with increasing circular aperture sizes: small, medium, and large (from left to right). **B** Average encoding performances across subjects over time for electrode Iz (top) and electrode Pz (bottom) for each stimulus size condition. Line colors indicate aperture condition (Center and Periphery), and line style indicates which encoding model the results belong to (Full and GCS). Asterisks at the bottom indicate significant correlations across subjects per condition for the Full model (permutation t-test). Asterisks at the top indicate the time point of the maximum correlation per participant, as well as the distribution across participants for the Center condition and Periphery condition with a black asterisk indicating a significant difference between the two conditions (Wilcoxon signed-rank test). **C** Average area under the curve of encoding performances (r) for each stimulus size condition and each encoding model for both aperture conditions: center (left) and periphery (right). Asterisks indicate significant differences (absence indicates non-significant comparison).

To directly verify these distinct temporal profiles, we performed a new, separate EEG experiment in which we explicitly stimulated foveal and peripheral regions only (see Methods section 4.1.2). The experimental paradigm was kept fixed to a large extent compared to the main experiment with the key manipulation that stimuli were presented using a circular aperture that varied in diameter to either exclude central information or exclude the directly inverse, peripheral information (see Fig 4A for examples of stimuli).

To test if directly stimulating distinct parts of the visual field resulted in earlier processing of peripheral than foveal information, we again built linearized encoding models to predict ERPs of held-out test images for data from each individual stimulus condition. Contrary to the main experiment, the input to the encoding model per stimulus condition is now the exact same as the stimuli seen by participants. Specifically, we tested: (i) whether we observe a temporal shift in the encoding performance of the Center stimulation condition compared to the Periphery stimulation condition, and (ii) whether the GCS transform improves predictions even when only selectively stimulating central or peripheral regions. Thus, we built two encoding models for each stimulus condition, one encoding model used the full feature map (“Full”) whereas the other used the GCS-transformed feature maps (“GCS”).

#### 2.2.1 Direct experimental evidence for temporally distinct processing of center and periphery.

Consistent with our previous modeling results (see Fig 3A), we find again a clear temporal difference between the Center and the Periphery stimulation condition for electrode Iz (see Fig 4B) for the Full encoding model when the circular aperture is smallest (earliest significant time points: 73 ms for Periphery, 85 ms for Center), and for electrode Pz for the small (65 ms for Periphery, 80 ms for Center) and medium aperture (59 ms for Periphery, 67 ms for Center). Statistical difference test reveal significant temporal differences between the maximum encoding performance between Center and Periphery conditions for both electrodes. For electrode Iz, encoding performance of the medium and large Periphery conditions is close to zero, indicating that peripheral information does not significantly drive the responses at electrode Iz.

Further, we find that the GCS model (mean encoding performance: 0.090 ± 0.163) only outperforms the Full model (mean encoding performance: 0.070 ± 0.141) in the large Center condition (Wilcoxon signed-rank test: W = 6, p = 2.734e-2), indicating that the differential modeling of foveal and peripheral information processing becomes important when approaching full-field visual stimulation (see Fig 4C). Overall, these results directly demonstrate temporally distinct profiles of processing central and peripheral information by selectively stimulating the visual field.

#### 2.2.2 Large-field stimulation is necessary to reveal center-periphery temporal profiles.

To test if spatial transformations of CNN feature maps aid encoding model performance in other datasets, we also ran a comparison of a standard (Full) encoding model and the GCS encoding model on a publicly available EEG dataset [17], containing responses for 10 subjects to 16,740 images from the THINGS dataset [55]. Unlike in our data, we see no improvement in encoding performance by including a GCS transformation at any electrode (see S2 Fig). We hypothesized that this difference could reflect a lack of peripheral stimulation in the dataset (image size 500x500 pixels, 7x7 degrees of visual angle), compared to our EEG recordings (image size 2155x1440 pixels, 50x29.5 degrees of visual angle).

**Fig 5:**
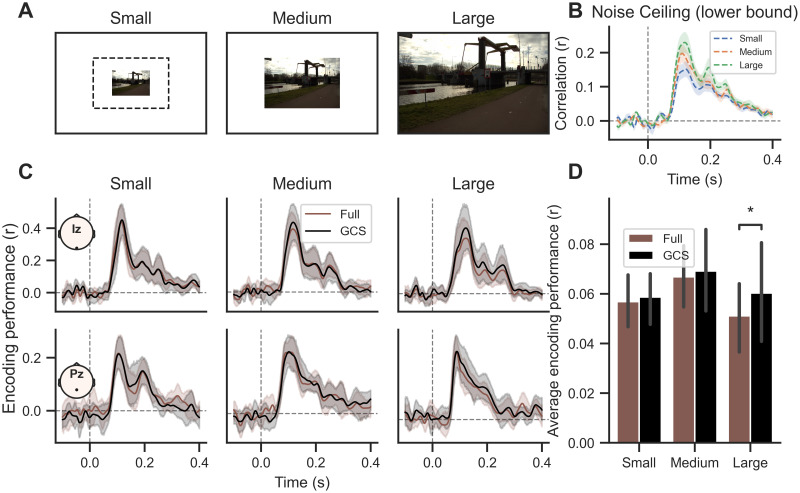
Signal-to-Noise ratio increases with size of visual stimulation. **A** Example stimuli for the three image size conditions in relative scale to each other. **B** Estimated lower bound of the noise ceiling across 10 subjects. Shaded areas show standard error, for each image size condition. **C** Cross-validated encoding performances (r) over time for Full and GCS encoding models of EEG signals from experimental runs with small, medium and large stimuli (see A). The average performance across subjects in shown for electrode Iz in the top row and for electrode Pz in the bottom row (axis insets with topoplots show the locations on the scalp). Colored shaded areas show standard error across subjects per encoding model per condition. **D** Bars show the mean and 95% CI across participants of the average encoding performance across time and electrodes per encoding model, per image size condition. Asterisks indicate statistically significant differences between Full and GCS models within a size condition (Wilcoxon signed-rank test, alpha = 0.05).

To test this hypothesis, we collected additional experimental data (see section 4.1.2) where we systematically decreased the stimulus extent compared to the main experiment (small, medium, large; see Fig 5A). This experiment is different from 1.2 as also the small and medium conditions show the full image but on a smaller area. Noise ceiling estimates of EEG responses from systematically varying stimulus extent show that the amount of stimulus-driven signal in the neural response increases for increasing stimulus size (see Figs 5B and S4 for ERPs for each conditions). Further, we find that there is indeed no benefit of GCS in the small condition, but a significant benefit for large stimuli (see Fig 5C + D: Wilcoxon signed-rank test for Full vs. GCS for small [W = 21, p = 5.566e-1], medium [W = 22, p = 6.25e-1], and large condition [W = 8, p = 4.883e-2]). These results suggest that the effects of retinal sampling on EEG responses are only revealed when using large-field stimulation that extends sufficiently into peripheral vision, thereby more closely approximating real-world vision.

### 2.3 Recovering center-periphery sampling from EEG

#### 2.3.1 Pixel-wise spatial contribution to explained variance of EEG.

We demonstrated distinct temporal profiles for foveal and peripheral processing by building encoding models that sample convolutional feature maps differentially. We confirmed these temporal differences experimentally using differential stimulation. Both these experiments however necessitated manual selection of parts of the visual field to sample or stimulate. In this last section, we ask: is it possible to reveal these distinct profiles without manually designating parts of the visual field as center and periphery. To this end, we introduce a new method that uses encoding model scores in combination with randomly sampling parts of CNN feature maps (see Methods section 4.2.2 for more details). By doing so, we can determine the contribution of each pixel in the CNN feature map (roughly corresponding to a region in the visual field) to the EEG encoding performance.

**Fig 6:**
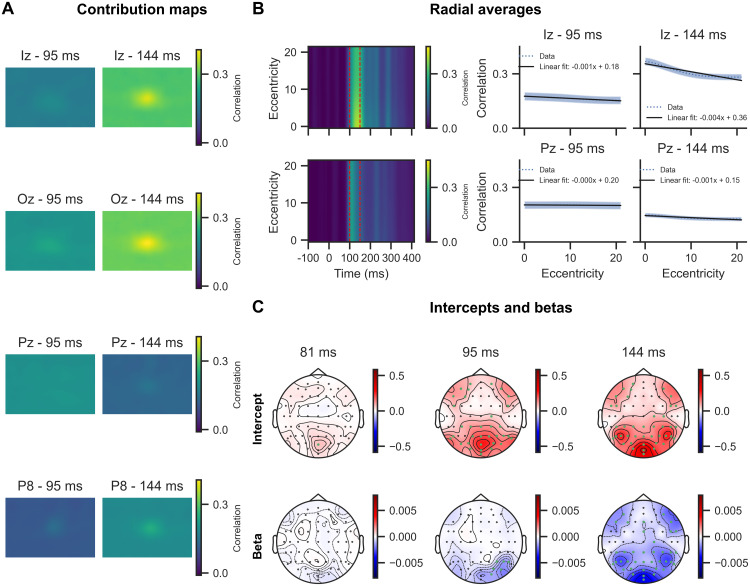
Fully data-driven recovery of spatial tuning in EEG recordings. **A** Average contribution maps across subjects representing the contribution of each spatial location in the visual field to the encoding performance. Contribution maps are unique per subject, electrode and time point and represent encoding performance (r) values. **B** Left column shows the radial averages over time for electrodes Iz and Pz. Middle and right columns show the radial average data (dashed line) per eccentricity for individual electrodes Iz and Pz at time point 95 ms and 144 ms after stimulus onset. Solid lines show the best linear fit (parameters are indicated in legends). A negative beta indicates that foveal regions contribute more to the encoding performance than peripheral regions. **C** Topoplots for individual time points (81 ms, 95 ms, 144 ms) show the average intercept (top row) and beta (bottom row) parameter across subjects per electrode. Green bold dots indicate statistical significance (permutation t-test with alpha = 0.05).

Fig 6A shows example contribution maps for electrodes Iz, Oz, Pz, and P8 for the early (95 ms) and late (144 ms) time points, showcasing electrodes with increased central contribution compared to peripheral (Iz and Oz) as well as equal contribution of central and peripheral regions (Pz, P8). These results, obtained in a fully data-driven way, qualitatively resemble the differential contributions of central and peripheral regions, which we have shown to drive responses at distinct points in time.

Next, in order to quantify the (non-)uniformity relative to the center per contribution map, we use radial averages of the contribution maps and the parameters (intercept and beta) of a linear regression model across eccentricity (see Fig 6B). We find that for electrode Iz at early time points (95 ms), there is a uniform contribution of all eccentricities to the overall encoding performance. However, for later time points (e.g., 144 ms) low eccentricities (closer to the fovea) contribute much more strongly to the encoding performance as indicated by the negative slope (beta = -0.004 at t = 144 ms). Contrarily, for electrode Pz, the contributions of different eccentricities remain uniform throughout the full time course.

We create a temporally-specific uniformity profile of the scalp, using the linear regression parameters per electrode. Fig 6C shows the average intercept (top row) and beta (bottom row) across subjects for each electrode for time points 81 ms, 95 ms, and 144 ms. Solid (green) dots indicate statistical significance (permutation test; 5000 iterations; alpha = 0.05). The earliest time point for which an electrode has a non-zero intercept is 81 ms for Pz, indicating the first time point after stimulus onset at which EEG data can be encoded. Crucially, at this time point, the beta parameter is still close to 0, indicating a uniform contribution across eccentricities. The earliest time point for which electrodes have a non-zero beta parameter is 95 ms, indicating more contribution of central locations compared to peripheral locations. The largest negative beta parameter occurs at 144 ms after stimulus onset at electrode Iz (beta = -0.004; intercept = 0.35).

Overall, we show that we can recover the differential weighting of specific locations underlying the encoding performance of each individual electrode and time point using iterative sampling, removing the necessity to specify center and periphery parts beforehand.

#### 2.3.2 Data-driven spatial weights yield best encoding performance.

The data-driven spatial contribution maps allow us to characterize each electrodes spatiotemporal tuning profile both qualitatively and quantitatively. However, to validate that these contribution maps do in fact closely resemble how central and peripheral information together drive the EEG responses, they should also lead to improved encoding performances.

To that end, we use the pixel-wise spatial contribution maps and apply them as a spatial weight to the CNN feature maps, per electrode and time point, fit a new encoding model (“Sampling”) and assess the cross-validated encoding performance on the held-out test set. We compare the encoding performance against the previously used full-field models (Full and GCS) and perform Wilcoxon signed-rank tests across subjects of the average encoding performance across electrodes and time points.

**Fig 7:**
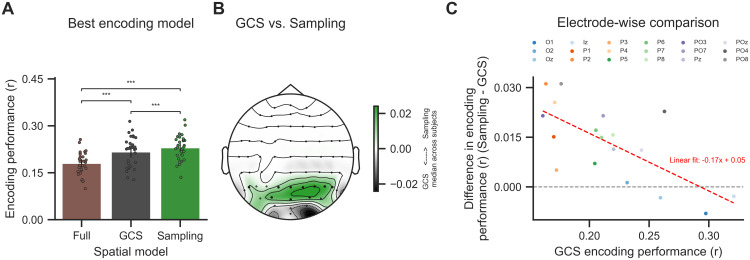
Spatially optimized encoding model outperform all other spatial sampling methods. **A** Encoding performance (r) is averaged across all time points and electrodes; bars and errorbars show the average and 95% CI across subjects, individual data points show individual subjects), for the Full, GCS, and Sampling encoding models. Asterisks between models indicate statistically significant differences across subjects (Wilcoxon signed-rank test, alpha = 0.05). **B** Topoplot shows the medians across subjects of the differences in time-averaged encoding performance (r) between GCS and Sampling models per electrode. Bold markers indicate statistically significant differences (Wilcoxon signed-rank test, alpha = 0.05). **C** Scatterplot shows the difference of the encoding performance between GCS and Sampling models per electrode (averaged across subjects) with the solid red line indicating the best linear fit.

This spatially-optimized Sampling model outperforms all other previously used models, including the Full (Wilcoxon signed-rank test: W = 0, p = 9.313e-10) and GCS (W = 54, p = 4.488e-5) model (see Fig 7A). Next, we performed a Wilcoxon signed-rank test between the GCS and spatially optimized time-averaged encoding performances across subjects per electrode and plot the median encoding performance differences and significant differences in Fig 7B: for most posterior electrodes the Sampling model significantly outperforms the GCS model. Only for the very occipital electrodes, the GCS model still performs on par with the Sampling model, indicating that the strong overrepresentation of central information by means of a real spatial transform, dominates the signal at this electrode. More so, for electrodes for which the GCS model performs generally better, the spatially optimized model shows less improvements compared to electrodes where the GCS model performs worse (see Fig 7C).

These results show that our iterative spatial sampling method is able to recover the electrode and time point specific spatial tuning, without assuming any prior knowledge. The recovered contribution maps offer a qualitative, interpretable description of spatial tuning and at the same time lead to the overall best encoding model, even compared to the previously tested GCS model.

## 3 Discussion

The human visual system is organized to a large extent with respect to the reference frame of the retina. Modeling this aspect of functional cortical organization has led to powerful forward models of neural responses to simple visual stimuli. Contrarily, encoding models built on feature maps from CNNs - that sample their visual input uniformly and thus lack retinotopic tuning - have become state-of-the-art in predicting neural responses to more complex, natural visual stimuli. Here, we explored how incorporating non-uniform spatial sampling into encoding models built on CNN feature maps influences their encoding performance of human EEG data. We found direct links between the spatial location of visual information (foveal vs. peripheral) and the temporal processing of that information, with peripheral information preceding foveal information encoding, in line with notions of coarse-to-fine hierarchies. Further, we used a novel, data-driven method to quantify the importance of each spatial location in the visual field for the encoding performance of each electrode and each point in time. For many electrodes, foveal regions contribute much more than peripheral regions, specifically later in time. Accordingly, we demonstrate that applying a retina-inspired transform on encoding model inputs, which weighs foveal information more strongly than peripheral information, uniformly improves encoding performance. Nevertheless, a selected number of electrodes prioritize non-central information, early in time, again in line with global-to-local processing frameworks of visual processing [43,44]. Collectively, these results highlight that even EEG signals that pool brain activity across large neural populations reflect differential spatial sampling in retinotopic visual cortex.

### 3.1 Distinct temporal profiles of peripheral and foveal processing

A global-to-local processing hierarchy is thought to be largely implemented through the parvocellular and magnocellular pathways. The response properties of the parvo and magno cells have been studied extensively and have been shown to underlie the specialization of processing of foveal and peripheral information [56]. The processing of incoming visual information is guided by saccades that move the fovea onto locations that should be sampled with high acuity [57]. Low-contrast and motion sensitive cells process peripheral information, thereby informing subsequent saccades while allowing for the detection of movement and approaching objects or agents. In addition, an important characteristic of the magnocellular pathway is the processing of *scene gist* [58]. Scene gist refers to the idea of processing the context of a scene [59] in very short time to facilitate self-orientation [60,61], processing action affordances [62,63], and setting up priors for fast object recognition [64].

Behavioral studies have shown the speed of scene gist processing [65] as well as the importance of peripheral stimulation for scene categorization tasks [36]. Further, EEG signals measured during RSVP experiments with humans have been shown to be highly predictable by image-computable scenes statistics [66]. Recently, [67] showed that training CNNs on blurred images, thereby approximating the peripheral percept, improves both behavioral and representational alignment with humans.

Our findings are consistent with the idea of a fast, coarse processing of peripheral information followed by a period during which the EEG signal is dominated by foveal stimulation, proposed in various global-to-local frameworks of visual processing [43,44,68]. By spatially selecting parts of the CNN feature maps, we show that information at the fixation within an image is most predictive of later time points at occipital electrodes. This signal is likely dominated by the activity of neurons in the primary visual cortex that have receptive field properties that correspond to a fine, foveal information processing [8].

By selecting parts of the CNN feature maps that correspond to peripheral scene information we showed that this information is being processed at early time points (as early as 75 ms; see Fig 2) and at more central and temporal electrodes. The temporal delay of the encoding of foveal information as well as the early encoding of peripheral information is in line with faster signal processing in (peripheral) magnocellular cells compared to (foveal) parvocellular cells. Our results provide new insights into how physiological differences such as RGC density and cellular processing speeds are reflected in EEG recordings. In sum, these results highlight a direct link between the spatial location of visual information and the temporal dynamics underlying its processing as measured using EEG.

### 3.2 CNNs as model organisms for visual spatial processing

While CNNs are often critiqued for their lack of biological plausibility [69], e.g., in terms of spatial tuning (lacking because of weight sharing), others have argued that CNNs should instead be seen as a model organism [70] that does not need to be a perfect model at every level of abstraction (e.g., simultaneous implementation of spiking behavior of individual units while also exhibiting modularity across different regions of the model [71,72]). Instead, CNNs can be seen as a model of overall (visual) information processing that can be used to compare specific hypotheses by testing alignment with brain data across multiple implementations of the CNN (e.g., with and without differential spatial sampling) [73,74].

Here, we provide evidence that this modeling approach is indeed valid and fruitful: next to modeling different spatial sampling methods on the feature maps of CNNs, we additionally collected experimental data of differential spatial stimulation. The temporal patterns revealed through the modeling of the processing of foveally and peripherally sampled information were the same as the ones revealed through stimulating foveal or peripheral regions. The modeling approach however, next to reducing experimental costs, also led to new predictions, that we empirically verified, following the neuroconnectionist research framework.

### 3.3 Studying real-world visual processing with large-field images

Historically, visual information processing in the human brain has been investigated using hand-crafted stimuli shown to participants in carefully designed neuroimaging experiments, to derive response properties (e.g., [6,75–78]). However, applying these methods to real-world visual stimuli is often hard or not possible, as discussed in debates around artificial vs. naturalistic stimulation in visual perception research [79–81]. It remains unclear whether the uncovered neural response properties also hold true during natural perception, and multiple studies have shown that neural responses can differ under stimulation with artificial vs. natural stimuli [48–52]. With the advent of CNNs that are intrinsically image-computable - meaning they can process arbitrary natural images - it has become possible to compare visual processing of natural images in humans with such models (e.g., [12,13,82]). We see computational (encoding) models as a promising solution to the challenge of balancing experimental control and ecological validity because they can be directly applied to neural data obtained from experiments using natural stimuli while allowing for a high degree of manipulability of their inputs, closely resembling how artificial stimulus manipulations are used in traditional experiments. However, only few efforts (e.g., [83]) have been made to extend this beyond the experimental setting of small stimulus sizes presented on low resolution screens during neuroimaging experiments. When using small size visual stimuli, only a small proportion of the visual field is stimulated. Stimulating only (para-)foveal regions does not require modeling of peripheral spatial sampling to accurately predict neural recordings (see Fig 5C). The prominent differences between the uniform sampling of CNNs and the non-uniform sampling of the human retina have therefore been found to be negligible. Here, we showed that in scenarios in which both foveal and peripheral regions are being stimulated, the differential sampling of foveal vs. peripheral information start to play a significant role for accurately predicting neural data (see Figs 4C and 5C-5D).

Moreover, we also showed that exclusively stimulating foveal regions reduces the signal-to-noise-ratio (SNR) of the EEG recordings compared to large-field stimulation (see Fig 5B). One possible explanation for these differences in SNR is that the lack of activation in the proportion of cortical volume that processes peripheral information increases the noise in the EEG signal when peripheral stimulation is absent. Interestingly, our results showed that the increased SNR in the EEG signal during large-field stimulation cannot be explained anymore by using the standard encoding model approach of mapping the entire feature map onto the neural data. Only when accounting for the differential spatial sampling of foveal and peripheral regions (e.g., using the GCS transform), is it possible to explain the additional explainable variance. Thus, while encoding models using CNN feature maps have been shown to yield high encoding performances when using small visual stimuli, this approach might not produce competitive encoding performances when using real-world naturalistic stimuli without accounting for the retinotopic tuning underlying the neural data.

### 3.4 Optimal spatial reweighting using retinal sampling

Next to work on modeling peripheral processing, attempts have been made to incorporate foveation into computational models of visual processing. Behavioral benefits have been observed by foveating training images of CNNs [84]. Bringing together both foveal and peripheral spatial sampling, [24] showed that a spatial reweighting of CNN training images that magnifies foveal information while degrading peripheral information yielded a hierarchy of receptive field properties that is similar to that of the human visual system. We found that the same spatial reweighting applied to pretrained CNN feature maps can improve encoding model performance, thereby predicting both early and late temporal dynamics of EEG signals well.

For certain electrodes, at central and temporal locations, we see that the periphery crop model explains unique variance that the GCS model does not capture (see Fig 3C). This shows that the GCS transform too strongly reduces the information density in the periphery and that in turn, peripheral information plays an essential role in the processing of natural scenes. An important parameter in the GCS transform is the magnification factor of foveal regions. Encoding analyses for different magnification factors showed that ERP responses at early time points were best explained using less drastic magnification while at later time points stronger magnification performed best, averaging out around the empirically-derived factor used by [24] of around 20 degrees (see S6 Fig). While this analysis yields a magnification parameter estimate for each electrode and time point, it is still limited to the specific geometric transformation implemented in the GCS algorithm. In comparison, our novel, fully data-driven approach finds the optimal spatial weighting for each electrode and time point independent of any geometric assumptions whilst also providing a quantitative parameter estimate that allows for qualitative inspection of the optimal spatial sampling - see the following section 3.5.

### 3.5 Recovering retinotopic information from EEG

Using a novel method that combines a cross-validated encoding model pipeline with randomly sampling parts of two-dimensional feature maps from CNNs, we showed that abundant information about the spatial tuning at each individual time point can be recovered from EEG signals. This analysis showed that a non-uniform sampling of the visual field is beneficial for encoding performances for many electrodes at many time points. However, it also revealed that specifically at early time points, more central and temporal electrodes show a uniform sampling across the visual field. This analysis thus yields both quantitative and qualitative evidence for a global-to-local processing hierarchy across space and time.

Overall, the proposed method is a promising candidate for revealing the spatial tuning of any neural data and its underlying (population of) neurons. While EEG signals are spatially pooled signals and thus offer little insight into the differences of response properties of nearby neural populations, we found that even for predicting this global signal, the retinotopic organization of visual cortex plays a crucial role. For other modalities (e.g., electrophysiology and fMRI), that reflect more fine-grained spatial differences, this method could be adapted without changes to recover the underlying spatial tuning (e.g., the population receptive field).

### 3.6 Using inductive biases to improve CNNs

State-of-the-art deep learning models trained on vast amounts of data outperform humans on various task, including object recognition. Thus, there is little room for improvements on pure performance by incorporating inductive biases into the CNN architecture. However, other aspects of deep learning model behavior, such as robustness to noise or learning efficiency, are potential candidates for further improvements through incorporating inductive biases that are known to contribute to how humans efficiently process their visual environment.

This could be achieved by drawing inspiration from the way humans evolved and learn. By subjecting models to constraints with regards to efficiency, number of parameters, or other architectural features, the model might develop specific inductive biases, such as a fovea-periphery distinction, purely from learning constraints. Recent work has started to explore whether there are benefits of inducing signatures of peripheral processing in CNNs; for example, training CNNs on foveated images has been shown to increase robustness to image manipulations [84] and training on blurred images (as a proxy for the peripheral percept) helps predict human fMRI and monkey electrophysiology data [67]. In addition, integrating foveal and peripheral sampling using spatial reweighting into a CNN leads to the emergence of a hierarchy of receptive field properties similar to that of the human visual system [24]. Yet, in all these examples, the changes to the model architecture are still performed manually. A more effective and plausible version would instead impose energy constraints on the distribution of input cells (here pixels) [85].

### 3.7 Temporal differences in encoding performance are independent of CNN architecture, training dataset, pretraining, and crop shape

The results presented in this manuscript are obtained using the features of an AlexNet, pretrained on ImageNet-1k object classification. To ensure that this specific choice of architecture did not influence the found patterns, we repeated our analysis for other pretrained architectures (ResNet18, ResNet50, ConvNeXt; [21,53]; see S7 Fig), for two CNNs trained on scene categorization on images from the Places365 dataset (Zhou et al. 2017), which contain more background information and are not focused on objects but rather on scene information (see S8 Fig), as well as for untrained versions of all four architectures (see S9 Fig). For each of these additional conditions, the temporal difference between peripheral and foveal encoding remained the same as for the pretrained AlexNet with the GCS model outperforming all other models. These findings highlight the robustness of the spatial information contained in EEG recordings and the independence of specific modeling choices. As expected, we do see that the overall encoding performance decreases substantially for untrained CNN versions compared to the pretrained versions. This shows that while task optimization through model training improves overall model-EEG alignment, the observed temporal processing signatures are consistently driven by spatial sampling, not feature tuning.

Last, we tested whether the choice of a specific crop shape (circular) influences the encoding performance or patterns found by repeating our encoding analysis using oval crops and rectangular crops (see S10 Fig). We find that the results are robust to these changes in crop shape, highlighting that the resulting temporal differences are caused by a specific spatial selection of information rather than a certain crop shape.

The observed robustness of the temporal differences in encoding performance against changes in model architecture are in line with previous work showing that model architecture is less important for reaching high encoding performance [86]. In contrast, our results show strong effects of spatial reweighting on the encoding performance, highlighting the substantial amount of retinotopic information reflected in the temporal profiles of human EEG recordings from natural scene viewing.

### 3.8 Limitations and suggestions for future work

The analysis of EEG recordings from the main experiment could have benefited from an increased number of repetitions per image condition as well as number of image conditions which have been shown to increase SNR (see e.g., [17]).

The employed method of retinal spatial reweighting (GCS) used here was originally introduced as a transform of the input to the CNN instead of to the CNN feature maps [24]. The input-level transformation would be a more biologically plausible implementation, posing processing within the CNN as cortical processing and the GCS as a retinal transform (prior to the cortex). However, applying the GCS transform on the input to the CNN with subsequent assessment of brain alignment would change the hypothesis that is being tested: does adding a retinal transform change the processing of a CNN such that it is more similar to that of the human visual system? This would put the CNN at the center of this hypothesis while our primary aim was to identify the temporal dynamics underlying the processing of foveal and peripheral information in human EEG.

Incorporating retinal sampling as part of the processing of CNNs would be an exciting way of moving towards more biologically plausible models of the human visual system [87]. Crucially, the GCS transform could be used to model not only foveal magnification, but also to simulate eye-movements by centering the GCS transform on an object instead of the center of the scene. While these experiments are out of scope for the current paper, we have made the OADS image dataset publicly available, such that future work can explore these directions.

Traditionally, retinotopic representations and temporal dynamics are studied separately, possibly because of assumptions that temporally-resolved recordings (such as EEG) are not subject to influences by retinotopic organization or that spatially-resolved recordings (such as fMRI) are not subject to influences by temporal sampling differences of, e.g., distinct cell types. Yet, there is clear behavioral evidence for differences in processing speeds for information presented at different eccentricities [88]. Recently, there have been efforts to combine these two different aspects in both fMRI [89] and EEG [90], and also using multimodal approaches [91]. We add to this evidence by showing that even EEG recordings that pool signals across the cortical surface contain signatures of retinotopic organization. This has important implications, even for studies using fMRI, for which it has been shown that there is systematic overlap between functionally-specialized and retinotopically-organized regions [92]: even high level aspects of visual processing such as functional specialization could be influenced by retinal processing that give rise to temporal differences in the processing of spatially distinct information. This requires future work to build more holistic models that integrate the temporal dynamics with the retinotopy of human visual cortex.

### 3.9 Conclusion

Overall, our results suggest that a fruitful approach of improving encoding models using CNN feature maps is the implementation of knowledge about the structural and functional organization of the human visual system into the encoding model. Specifically, we address a well-known issue regarding the biological plausibility of the spatial sampling of CNNs by incorporating non-uniform spatial sampling into encoding models of human EEG data. We not only showed that encoding performance can be significantly improved by taking these structural constraints into account, but that is it necessary to maintain high encoding performance when visual stimulation becomes more natural. Last, using a novel, fully data-driven method based on random sampling of CNN features, we elucidated the precise temporal dynamics of peripheral and foveal processing, providing direct evidence for global-to-local processing theories of naturalistic visual processing.

## 4 Methods

### 4.1 Data collection

#### 4.1.1 Main experiment.

##### Ethics statement

The Ethics Review Board of the University of Amsterdam approved the experiment and all participants gave written informed consent before participation and were rewarded with study credits.

##### Subjects

We collected human EEG data from 31 participants (mean age 22.0 years, SD = 3; 18 female, 7 male, 6 other) with normal or corrected-to-normal vision in one session. Four participants (not included above) were excluded from analysis because their recordings were incomplete.

##### Stimulus set

To study differential foveal and peripheral processing in naturalistic settings, large, high-quality images are needed that allow for large-field visual stimulation. We used the Open Amsterdam Data Set (OADS), a new stimulus set consisting of 6130 ultra-high resolution outdoor scenes for both human data collection and computational modeling of cortical visual processing. OADS images consist of photographs of diverse street scenes taken in the city of Amsterdam, The Netherlands, have an original resolution of 5468x3672 pixels, and are stored in an uncompressed format. Stimuli contain images of streets, buildings, parks, roads with and without cars, bicycles, pedestrians, and other, commonly found, objects of urban environments. Additionally, a set of 27 indoor scene images with the same resolution depicting office scenes served as target images for the behavioral task in the EEG experiment (see below).

##### Experimental procedure

Participants were presented with OADS scene stimuli downsampled to a resolution of 2155x1440 pixels, to match the resolution of the monitor, using an RSVP paradigm [93–95]. Stimuli were shown on a 24-inch monitor with a resolution of 2560x1440 pixels and a refresh rate of 144 Hz. Subjects were seated 63.5 cm from the monitor such that stimuli spanned 50x29.5 degrees of visual angle, and a head rest was used to stabilize their head position.

The stimulus set used in this experiment consisted of 4680 randomly selected images (of the total 6130) of which every subject was presented with a subset of 702 images to allow for multiple repetitions per images while maintaining a reasonable session duration (an overview of the overlap between stimuli seen by subjects is shown in S1 Fig). Per participant, 1/3 of all images were repeated 10 times throughout the entire experiment while the other 2/3 were repeated 5 times. The resulting EEG data were averaged across repetitions to increase the Signal-To-Noise-Ratio (SNR) per image. Following standards in the linearized encoding literature (e.g., [17]), we created a low-SNR training image set to fit the encoding model by averaging across the 5 repetitions stimuli and a high-SNR test image set by averaging across the 10 repetitions stimuli.

A trial consisted of an RSVP [93] stream of a series of 20 images of either only outdoor scenes (non-target trial) or 19 outdoor scenes and one indoor scene (target trial), with 50% of the trials being target-trials. Indoor scenes, if present, were presented at a random position in the series of 20 images. Participants were instructed to perform an indoor scene detection task by pressing either ’J’ for a target trial or ’K’ for a non-target trial on a keyboard, at the end of the trial. Each trial started with a 3500 ms gray screen and a red fixation cross in the center of the screen which was continuously presented during the entire trial. Next, each of the 20 stimulus images were presented for 100 ms followed by a 300 ms gray screen each. At the end of the trial, subjects had 3500 ms to respond, and were instructed to blink before the start of the next trial. Each participant performed 240 trials. The session was divided into 8 blocks of 30 trials, each followed by an enforced break of at least 30 seconds with self-paced continuation. Stimuli were presented and behavioural data collected using *PsychoPy* [96].

##### EEG acquisition and preprocessing

EEG data was collected using a Biosemi 64-channel ActiveTwo EEG system (Biosemi Instrumentation) with an extended 10–20 layout, modified with two additional occipital electrodes (I1 and I2, while removing electrodes F5 and F6). Eye movements were monitored with electro-oculograms (EOGs). EEG recording was followed by offline re-referencing to external electrodes placed on the earlobes. Resulting EEG data was pre-processed, according to the standard pipeline in our lab (e.g., [97]) using the MNE software package [98]: high-pass filter at 0.1 Hz (12 dB/octave) and a low-pass filter at 30.0 Hz (24 dB/octave) followed by two notch filters at 50 and 60 Hz; automatic removal of deflections >300 μV; epoch segmentation in -100–400 ms from stimulus onset; occular correction using the EOG electrodes [99]; baseline correction between -100 and 0 ms; and conversion to Current Source Density [100]. The resulting event-related potentials (ERPs) were averaged across image repetitions, thus resulting in an ERP specific to each subject, electrode and image. ERPs to target (indoor) images were excluded from data analysis.

#### 4.1.2 Additional experiments.

##### Ethics statement

The Ethics Review Board of the University of Amsterdam approved the experiment and all participants gave written informed consent before participation and were rewarded with 180€.

##### Subjects

Human EEG data was collected from 10 new participants (mean age 22.6 years, SD = 3.7; 5 female, 5 male) in 6 sessions.

##### Stimulus set

We used a random subset of 697 outdoor scenes from the same stimulus set described in section 4.1.1. Additionally, a set of 35 indoor scene images served as target images in the additional experiments.

##### Additional stimuli 1 - central and peripheral stimulation

To test whether selective stimulation of central and peripheral visual field regions elicits temporally distinct ERP responses, we created stimulus version, where either only central information is kept while peripheral information is discarded, or vice versa, keeping peripheral information and discarding central information. For each of the 697 images, we created 3x2 additional stimulus versions. Using a circular aperture of increasing diameter (3°, 11°, and 20°), we created a center crop version that removed everything outside of the circular aperture (thus making it the same gray as the screen background) and a periphery crop version that removed everything inside the circular aperture (see Fig 4A). For each image and each of the three aperture sizes there are thus 2 versions, one containing only the parts of the image in the center, and one containing only the parts of the image in the periphery, but not in the center.

##### Additional stimuli 2 - stimulus size

Here, we used the original, intact stimuli, but presented them centrally at different sizes 2155x1440 pixels (large), 1079x720 pixels (medium), and 529x360 pixels (small). Thus, for each original image, 9 different stimulus conditions were presented (6 for center/periphery, 3 for sizes).

##### Experimental procedure

This experiment was conducted using the same experimental paradigm as the main experiment (see section 3.1.1). The following details differed in the additional experiment: all participants saw the same set of 6273 (697 images x 9 conditions) stimuli. As before, separate training and test sets were created by repeating now 35% of all stimuli 10 times while the other 65% were repeated 5 times.

Each participant performed 2706 trials (451 per session). Similar to the main experiment, a series of 20 images was presented in each trial. In contrast to the main experiment, 50% of trials contained a single indoor image, 25% of trials contained two indoor images, and 12.5% contained three images. Trials with either one or three indoor images were defined as target trials. This change compared to the main experiment was introduced to enhance attention, specifically because 6 sessions were performed.

##### EEG acquisition and preprocessing

EEG signals were recorded using the same setup as outlined in the main experiment with the difference of electrodes F5 and F6 not being replaced by electrodes I1 and I2. Raw EEG data was preprocessed using the same pipeline as outlined in section 4.1.1.

##### Eye-tracking data acquisition

To allow a control analysis excluding potential confounding influences on ERP responses between stimulus conditions due to eye movements (e.g., more difficulty maintaining fixation for center vs. periphery stimuli), we recorded eye-tracking data using an Eye Link 1000 for 6/10 participants (for more information on the analysis see S1 Text and S5 Fig).

### 4.2 Computational modeling

#### 4.2.1 Regression on trial-averaged ERPs.

##### EEG encoding models

We built linearized encoding models to map convolutional features from a task-optimized CNN (alexnet [19] pretrained on ImageNet [101] 1000-way object classification), onto the ERP amplitude for each subject, electrode and time point. To do so, we passed the intact OADS images through the CNN and extracted the feature maps from all max pooling layers (features.2, features.5, and features.12) using *PyTorch* [102]. Layers were selected by grouping them into “processing block” that applied a convolution, a non-linearity and a max-pooling, of which the output was then extracted. After feature extraction, we applied several spatial feature selections and fitted linear regression models for each selection individually.

##### Spatial feature selection

We applied spatial feature selection on each extracted convolutional feature map. Feature selection was done in one of the following ways:

**Center** - a circular crop of 0.5% of the area of the full 2D feature map was taken at the center of each feature map. The rest of the feature map was discarded. The absolute diameter of the crop depended on the layer and was 9x9 pixels for layer1 (64 channels), 3x3 pixels for layer2 (192 channels), and 1x1 pixels for layer3 (256 channels).

**Periphery** - the central 0.5% of the area of the full feature map was removed from the center of each feature map. This was thus the inverse selection of the center crop and maintains 99.5% of the total feature map.

**Ganglion cell sampling** (GCS) [24] - a retinal ganglion cell transform was applied to the full feature map. The GCS transform applies a magnification to central pixels and reduces the spatial weighting of peripheral pixels. The output of the transform is always square with the length of each side being the maximum of the input side lengths. The default parameters reported in the original paper were used with most importantly the magnification factor (“foveal size”) set to 20° (originating from empirical measurements of the density of human retinal ganglion cells).

**Full (baseline)** Finally, as a baseline, we simply used the full feature map as input for the encoding model.

For each of these 4 different feature selection methods, we fitted a separate linearized encoding model, as described in the following.

##### Linearized encoding model

We built linearized encoding models, consisting of a principal component analysis (PCA) and a linear (ordinary least squares) regression. As described above, the stimuli used during the EEG experiment were divided into two sets: a low-SNR set (images with 5 repetitions) and a high-SNR set (images with 10 repetitions). Features of the stimuli from the low-SNR set were used to fit the linearized encoding model (training set) while the features from the stimuli from the high-SNR set were used to evaluate performance and generalizability (test set).

For every model instance, we flattened the selected features per stimulus in the training set across all layers and performed a PCA across these features (the retained variance per model can be found in S12 Fig). The projections per stimulus of the first 100 PCs formed the design matrix. The ERP amplitudes for every subject, electrode, and time point were then regressed onto the design matrix. On the selected features of the stimuli of the test set, we applied the same PC projections and then used the fitted linear regression model to predict the ERP amplitude for the corresponding subject, electrode, and time point. For each model, the cross-validated encoding performance was quantified by calculating the Pearson’s correlation coefficient (r) between predicted and real ERP amplitude across test images for each subject, electrode, and time point.

##### Statistical significance

For each model’s encoding performance, we performed a one-sample permutation t-test against 0 across subjects for each electrode and time point using the *MNE* software package [98]. We applied false-discovery-rate (FDR) [103] correction across all time points per electrode and indicated statistical significance for alpha = 0.05 if not stated otherwise.

Additionally, for pairs of encoding models we compared encoding performances to determine the best performing model at each electrode and time point. We performed a Wilcoxon signed-rank test across subjects of the encoding model performances per electrode and per time point using the *scipy* software package [104]. We applied FDR-correction across all test results and indicated statistical significance for alpha = 0.05 if not stated otherwise.

Further, to test whether encoding models show differing temporal dynamics, we compare the distribution of time points of the maximum encoding performance per participants for individual electrodes for a given pair of encoding models. We performed a Wilcoxon signed-rank test between encoding models across subjects with alpha = 0.05, to test whether there is significant difference between the time points of maximum encoding performance.

##### Noise ceiling calculation

We calculated the noise ceiling, i.e., the theoretical maximum of explainable variance of the ERP amplitude across repetitions of stimuli, per subject, electrode and time point using the same method as described in [17]. Specifically, we split the ERP amplitudes across ten repetitions for the test set into two non-overlapping splits of 5 repetitions each. For each subject, electrode and time point, we estimated the lower bound of the noise ceiling as the correlation between the average of the first split of 5 repetitions and the average of the second split of 5 repetitions, and the upper bound as the correlation between the average of the first split of 5 repetitions and the average of all 10 repetitions. Noise ceilings for the additional experiments per condition are omitted in Fig 4 for clarity but can be found in S3 Fig together with the mean ERPs per condition.

##### Partial correlation

To identify the unique contributions of each spatial selection method, we calculated partial correlations using the *pingouin* software package [105]. For pairs of encoding models, we calculate partial correlations between each model’s predictions and the observed ERP amplitudes on the test set while regressing out the variance predicted by the other model, for each subject, electrode, and time point. We report partial correlations for selected electrodes, over time for Center vs. Periphery, Center vs. GCS, Periphery vs. GCS, and Full vs. GCS model pairs.

##### Selection of electrodes and time points

The large number of data points and ensuing statistical results (31 subjects x 64 electrodes x 513 time point x 4 encoding models) required selection of representative results. For Figs 2–5 two electrodes have been chosen to represent occipital electrodes (Iz) and more central electrodes (Pz). Additionally, for reporting topoplots in Fig 2 we selected three time points as representatives for early (95 ms after stimulus onset), intermediate (119 ms), and late (144 ms) time points. In Fig 7, we plot both the average across the 19 posterior electrodes (O1, Oz, Iz, O2, PO7, PO3, POz, Pz, PO4, PO8, P7, P5, P3, P1, Pz, P2, P4, P6, P8) (in accordance with [17]) and across time in panel A, as well as a topoplot showing the average across time per electrode.

#### 4.2.2 Pixel-wise contribution to encoding performance through random spatial sampling.

##### Iterative random sampling

To estimate the spatial tuning underlying the EEG data, we performed iterative sampling of random locations of the CNN feature map and quantified the location’s impact on the encoding performance, when only using information from that location. In each iteration, the visual information of a random combination of pixels was kept intact, while all other pixels in the feature map were set to 0. More specifically, in each iteration, we created a two-dimensional binary mask of the same size as the feature maps from layer1, ultimately containing 10 patches of 4x4 pixels. The mask was iteratively created by drawing a row index and a column index from a uniform distribution, then creating a 4x4 patch around those coordinates in the mask with the value 1, if the patch did not overlap with an already existing patch for the current iteration. For subsequent layers, the same mask was resized and reused to ensure overlapping spatial locations across layers. The extracted features maps of the training set were then multiplied with their respective mask per layer. Per layer, the features of all channels and all images were multiplied with the same mask.

The resulting masked feature maps were then projected into a lower-dimensional space using the pre-fitted PCA of the full feature condition. Subsequently, we used the pre-fitted linear regression model of the full feature condition to obtain predictions on the train set. We then computed the encoding performance (r) per electrode and time point for these predictions as the correlation with the measured data and assigned the encoding performance score to all pixels that were sampled in this iteration. The resulting encoding scores, obtained exclusively on the training set, across 22750 iterations were subsequently averaged per pixel. We refer to the resulting two-dimensional map containing the average encoding score per pixel as the *pixel-wise contribution map* for each electrode, time point, and subject.

##### Using pixel-wise contribution maps as spatial weights1

After estimating the pixel-wise contribution maps exclusively on the training set, we used them for a new strategy of spatial selection in the encoding model, namely as a form of multiplicative spatial weight. For each image, we applied the pixel-wise contribution maps, after min-max-scaling as a multiplicative weight to each feature map of the three previously used layers, for each electrode, time point and subject, separately. As previously described, we then fitted a PCA and linear regression on the features of the training set and compared the resulting cross-validated encoding performances to those of the previously used encoding models using a Wilcoxon signed-rank test across subjects (alpha = 0.05).

##### Characterizing the spatial uniformity of pixel-wise contribution maps

To quantify the (non-)uniformity of the pixel-wise contribution maps with regards to the fovea, we computed radial averages (i.e., the average of concentric circles moving away from the center) yielding the average correlation value at any given eccentricity. Subsequently, we fit a linear regression for each participant, electrode, and time point across eccentricities, yielding an intercept and a slope parameter. These parameters can be used to characterize the differences between central (low eccentricities) and peripheral (high eccentricities) locations: a slope parameter close to 0 implies that there is little to no difference between central and peripheral regions while a negative slope implies that central regions on average contribute more to the encoding performance than peripheral regions and vice versa for a positive slope. We perform a one-sample t-tests for each electrode and time point for the slope and intercept parameter across subjects against 0 and report the outcomes per electrode in Fig 6C.

## Supporting information

S1 Fig Experimental setup. Overview of the shared images between subjects. Pairs of subjects could either see all the same images, only the same test images, only the same training images, or see a fully distinct set of images.
(TIF)

S2 Fig THINGS EEG dataset - GCS model performs equally well as full model on large public EEG dataset [17]. **a)** Mean encoding model performance across subject for full (brown) and GCS (black) model over time for four representative electrodes over time. Shaded areas indicate 95% confidence interval per time point across subjects. At no time point for the selected electrodes does there appear to be an advantage of either model over the respective other. **b)** Mean encoding model performance across electrodes per subject for full (brown) and GCS (black) models averaged across all time point after stimulus onset. Dashed lines indicate subject average per encoding model. It seems as if no consistent difference between encoding model performances appear across subjects.
(TIF)

S3 Fig Noise ceilings for center and periphery stimulation conditions - Distinct neural signatures of central vs. peripheral stimulation. **a)** Example stimuli for central vs. peripheral stimulation using a circular aperture increasing size, from left to right. **b)** Average ERPs across subjects for center (orange) and periphery (blue) conditions for increasing aperture sizes. ERPs are higher for peripheral stimulation at time point between 100 and 150 ms after stimulus onset. **c)** Noise ceiling lower bound estimate averaged across subjects for center (orange) and periphery (blue) conditions for increasing aperture sizes. For small apertures, peripheral stimulation yields a higher SNR earlier in time compared to central stimulation. With increasing aperture size, SNR for central stimulation increases while SNR for peripheral stimulation decreases.
(TIF)

S4 Fig ERPs for size stimulation conditions. **a)** Example stimuli for small, medium, and large stimulus sizes, from left to right. **b)** Average ERPs across 16 posterior electrodes for each subject individually (colored lines) plus subject averaged (thick black line) for increasing stimulus size conditions.
(TIF)

S1 Text Eye-tracking data analysis. (DOCX)

S5 Fig Eye-tracking data analysis. Comparison between encoding performances of Full model for electrodes Iz (top row) and Pz (bottom row) for EEG data split into two set: one set consists only of the trial-averaged ERPs for trials during which participants did not perform any saccades (“Fixations”) while the second set consists of exactly those trials during which participants did perform a saccade. We find that the temporal difference between the Center and Periphery conditions is drastically reduced for electrode Pz even disappears for electrode Iz, showing that the observed temporal differences cannot be fully explained by the saccade behavior only.
(TIF)

S6 Fig GCS parameter comparison. We repeated the encoding analysis using the GCS model multiple times while varying the magnification factor. **a)** Average encoding performance (r) across participants for the GCS model using different magnification factors. **b)** Average magnification factor across participants yielding the highest encoding performance per time point, per electrode. Time points and electrodes for which the encoding performances across subjects were not significantly above 0 were removed (blank entries). **c)** Same data as in b), but averaged across groups of electrodes (colored topoplot on the left indicates the location of electrode groups).
(TIF)

S7 Fig CNN architecture comparison. Average encoding performance (r) across participants of the four models (Full, Center, Periphery, GCS) based on features of different pretrained CNN architectures: ResNet18, ResNet50, ConvNeXT. The temporal delay between the encoding of peripheral and central information persists when using features from other CNN architectures.
(TIF)

S8 Fig CNN pretraining comparison. Average encoding performance (r) across participants of the four models (Full, Center, Periphery, GCS) based on features of different CNN architectures trained on datasets other than ImageNet: AlexNet and ResNet50 trained on Places365. The temporal delay between the encoding of peripheral and central information persists when using features from CNNs trained on a dataset and task (scene categorization) that is centered on scene information instead of on object information.
(TIF)

S9 Fig CNN trained-untrained comparison. Average encoding performance (r) across participants of the four models (Full, Center, Periphery, GCS) based on features of different untrained CNN architectures. The temporal delay between the encoding of peripheral and central information persists when using features from untrained CNNs.
(TIF)

S10 Fig Crop type comparison. Average encoding performance (r) across participants of the Center and Periphery models using different crop types: instead of using circular crop (see Fig 2), we used oval crops or rectangular crops, both with a horizontal radius that is larger than the vertical radius.
(TIF)

S11 Fig Encoding performance for electrode-groups. Average encoding performance (r) across participants for the four encoding models (Full, Center, Periphery, GCS) averaged across groups of electrodes. Topoplot insets indicate the location of the electrode included per group and names above follow the same order, from left to right. The temporal delay between the encoding of central and peripheral information persists after averaging the results across spatially-adjacent electrode groups.
(TIF)

S12 Fig Comparison of retained variance by PCA on feature maps after spatial sampling. Average explained variance (summed across all 100 components) across participants for the four encoding models (Full, Center, Periphery, GCS) of the PCA fitted on the feature maps after applying the respective spatial sampling.
(TIF)
