# Supplementary figures and images for: Linking retinal sampling in neural encoding models to temporal profiles of visual processing in humans
Source: PLoS Comput Biol. 2026 Jun 30;22(6):e1014371. doi: 10.1371/journal.pcbi.1014371 (PMC13340806; doi:10.1371/journal.pcbi.1014371)

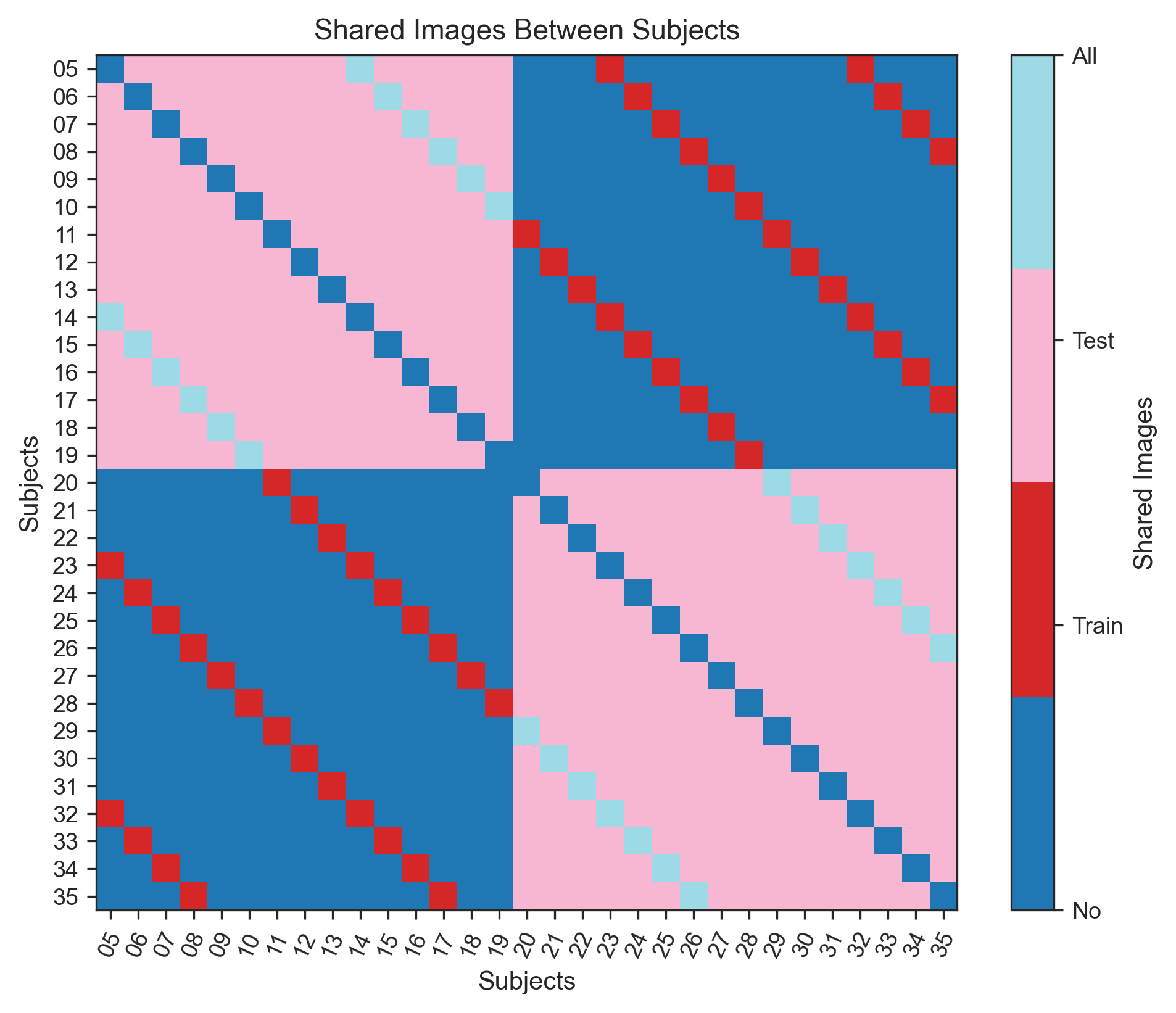

Supplement: S1 Fig — Overview of the shared images between subjects. Pairs of subjects could either see all the same images, only the same test images, only the same training images, or see a fully distinct set of images. (TIF) [file pcbi.1014371.s001.tif]

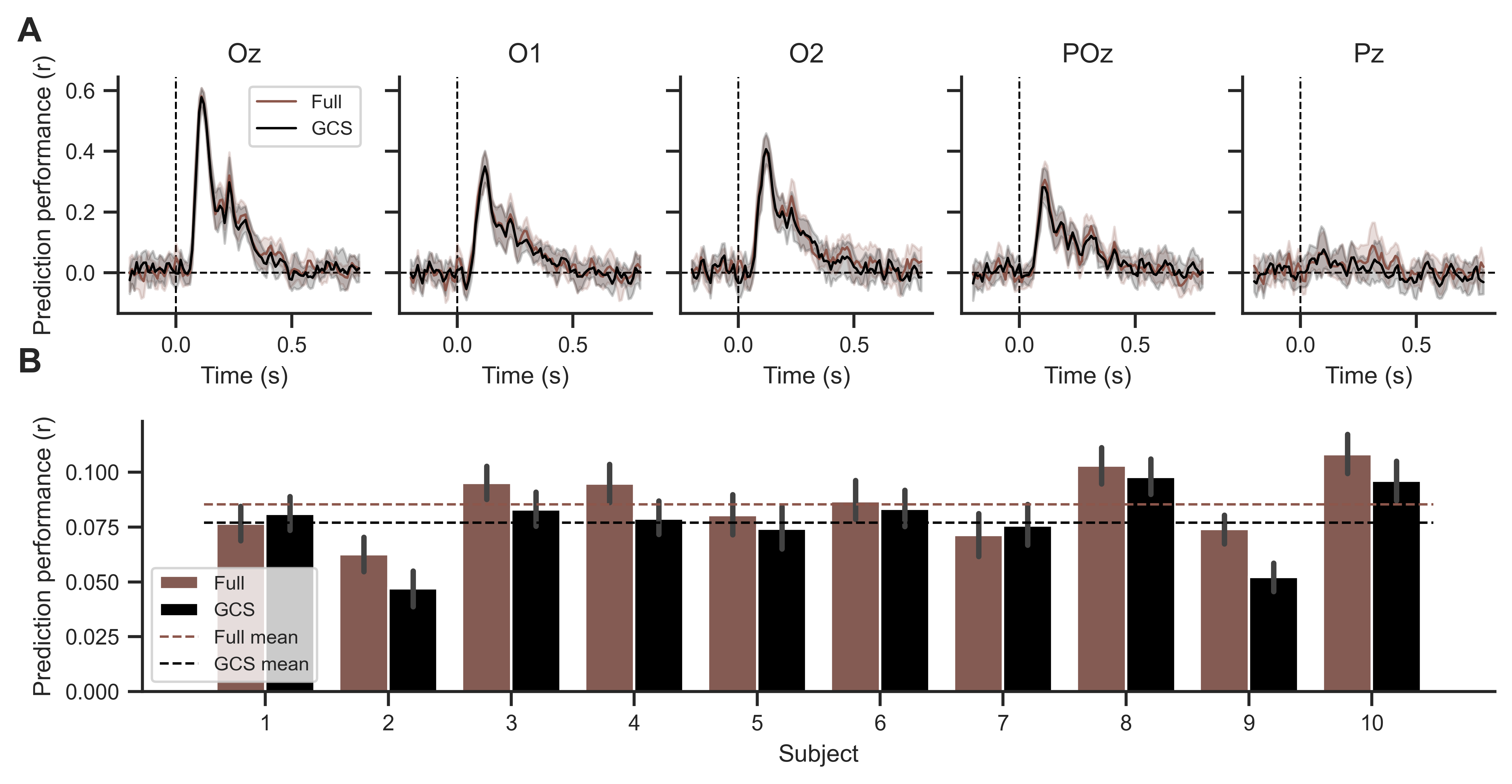

Supplement: S2 Fig — a) Mean encoding model performance across subject for full (brown) and GCS (black) model over time for four representative electrodes over time. Shaded areas indicate 95% confidence interval per time point across subjects. At no time point for the selected electrodes does there appear to be an advantage of either model over the respective other. b) Mean encoding model performance across electrodes per subject for full (brown) and GCS (black) models averaged across all time point after stimulus onset. Dashed lines indicate subject average per encoding model. It seems as if no consistent difference between encoding model performances appear across subjects. (TIF) [file pcbi.1014371.s002.tif]

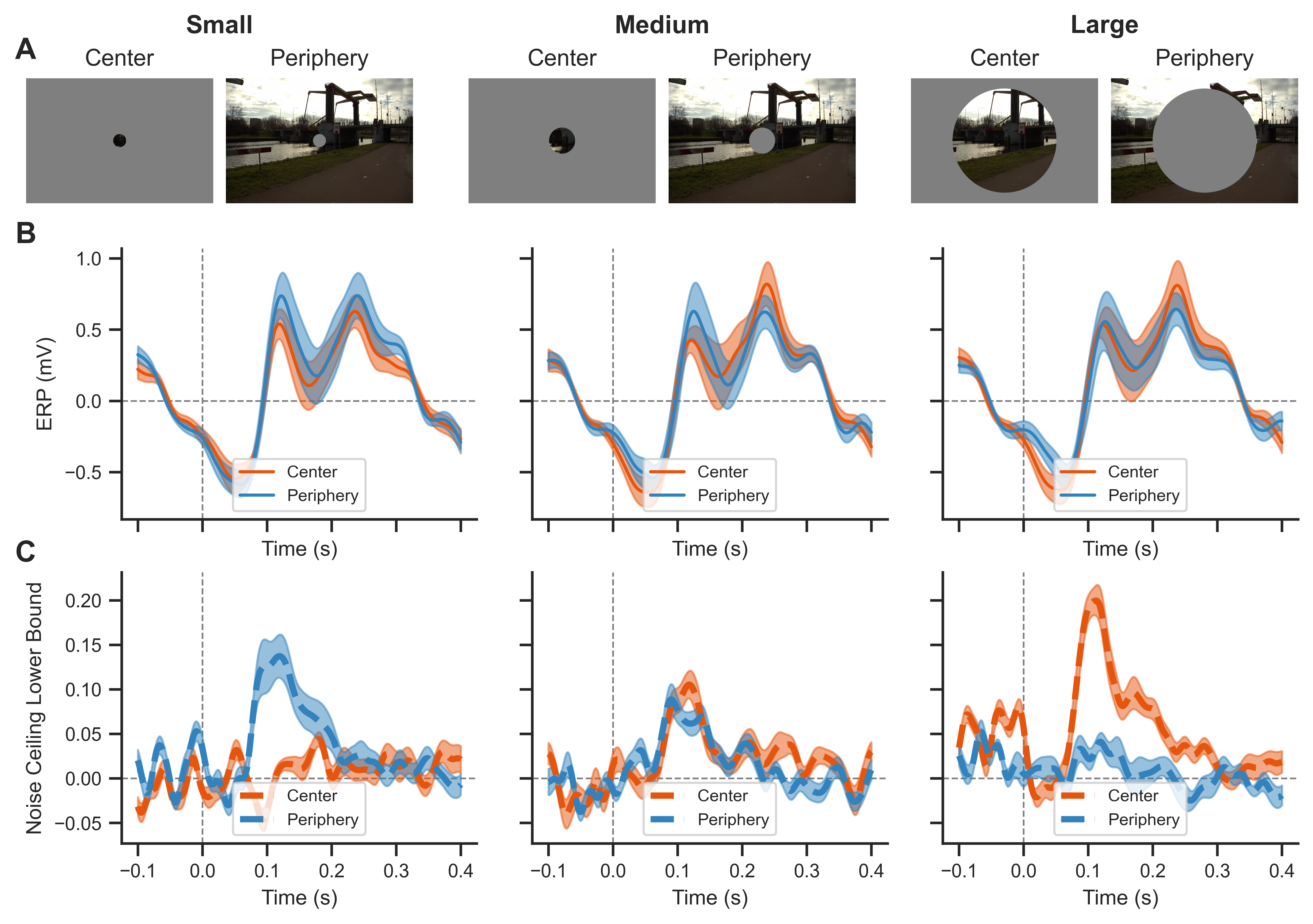

Supplement: S3 Fig — a) Example stimuli for central vs. peripheral stimulation using a circular aperture increasing size, from left to right. b) Average ERPs across subjects for center (orange) and periphery (blue) conditions for increasing aperture sizes. ERPs are higher for peripheral stimulation at time point between 100 and 150 ms after stimulus onset. c) Noise ceiling lower bound estimate averaged across subjects for center (orange) and periphery (blue) conditions for increasing aperture sizes. For small apertures, peripheral stimulation yields a higher SNR earlier in time compared to central stimulation. With increasing aperture size, SNR for central stimulation increases while SNR for peripheral stimulation decreases. (TIF) [file pcbi.1014371.s003.tif]

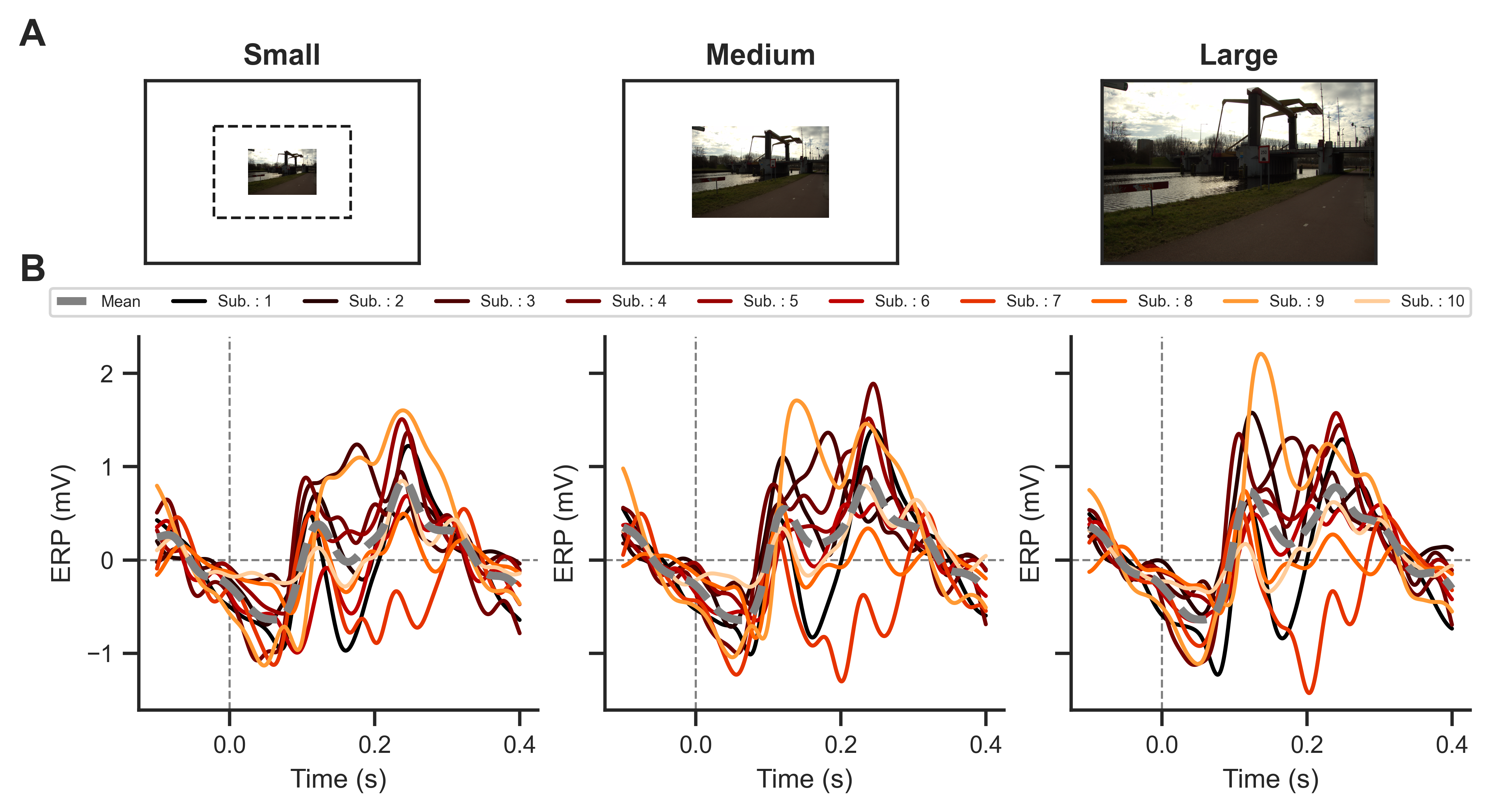

Supplement: S4 Fig — a) Example stimuli for small, medium, and large stimulus sizes, from left to right. b) Average ERPs across 16 posterior electrodes for each subject individually (colored lines) plus subject averaged (thick black line) for increasing stimulus size conditions. (TIF) [file pcbi.1014371.s004.tif]

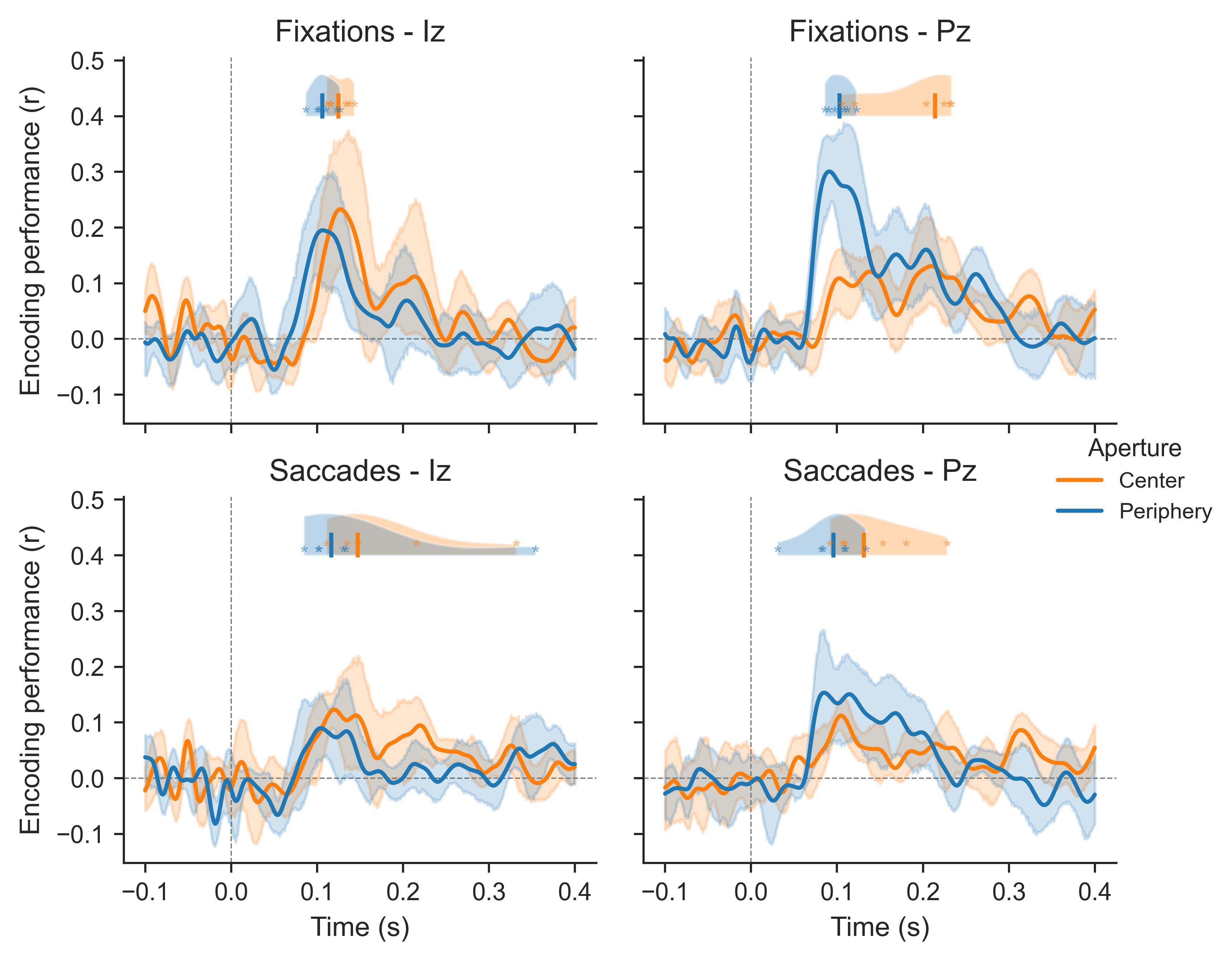

Supplement: S5 Fig — Comparison between encoding performances of Full model for electrodes Iz (top row) and Pz (bottom row) for EEG data split into two set: one set consists only of the trial-averaged ERPs for trials during which participants did not perform any saccades (“Fixations”) while the second set consists of exactly those trials during which participants did perform a saccade. We find that the temporal difference between the Center and Periphery conditions is drastically reduced for electrode Pz even disappears for electrode Iz, showing that the observed temporal differences cannot be fully explained by the saccade behavior only. (TIF) [file pcbi.1014371.s006.tif]

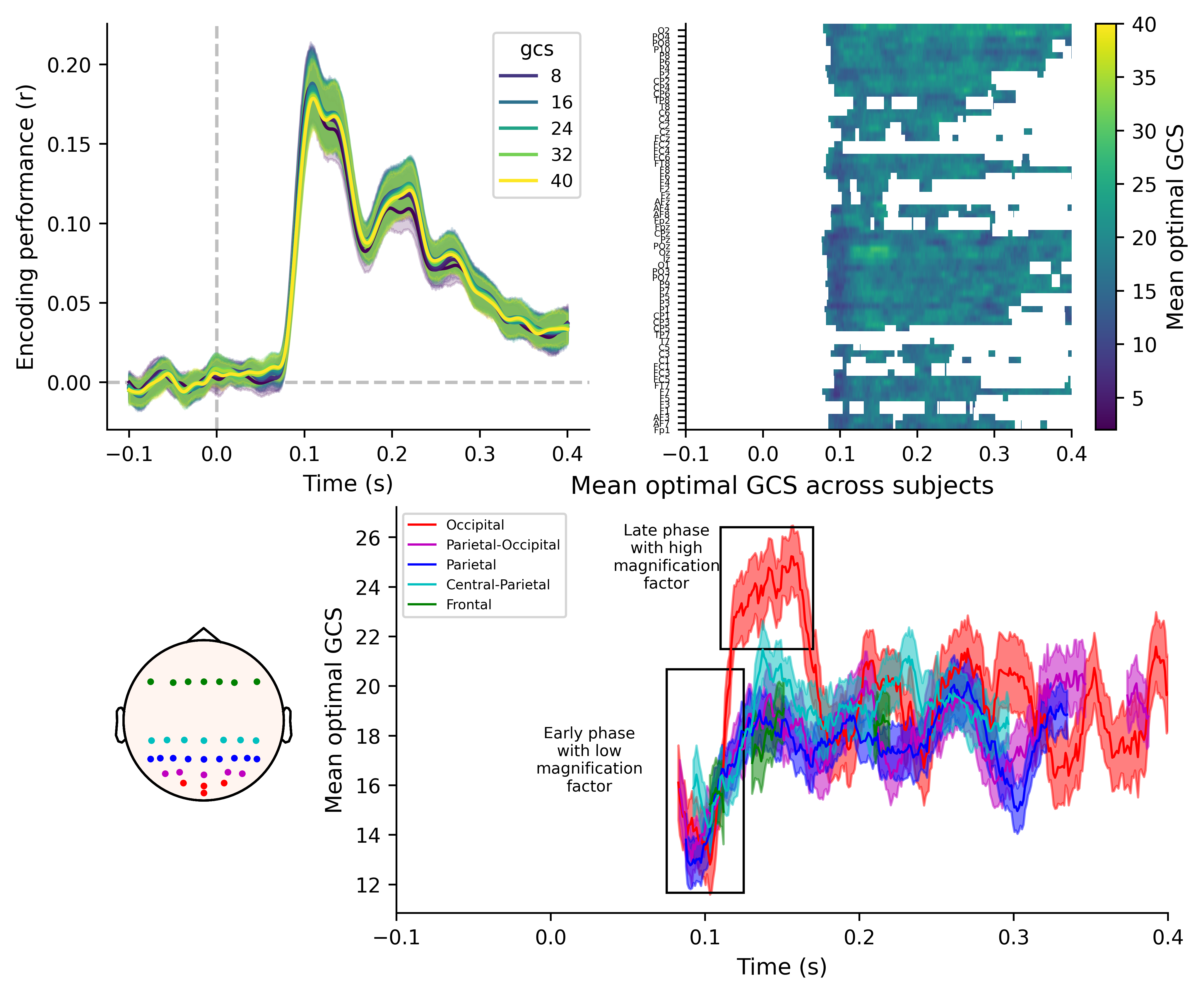

Supplement: S6 Fig — We repeated the encoding analysis using the GCS model multiple times while varying the magnification factor. a) Average encoding performance (r) across participants for the GCS model using different magnification factors. b) Average magnification factor across participants yielding the highest encoding performance per time point, per electrode. Time points and electrodes for which the encoding performances across subjects were not significantly above 0 were removed (blank entries). c) Same data as in b), but averaged across groups of electrodes (colored topoplot on the left indicates the location of electrode groups). (TIF) [file pcbi.1014371.s007.tif]

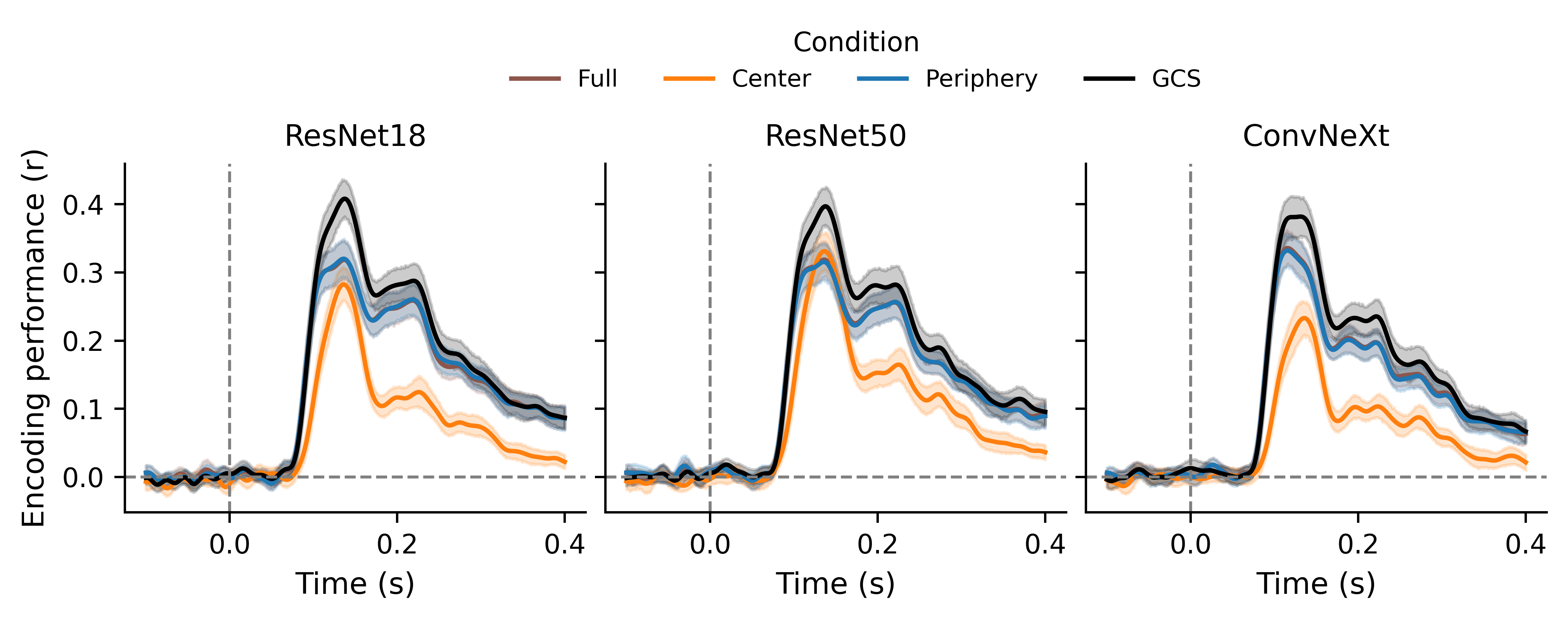

Supplement: S7 Fig — Average encoding performance (r) across participants of the four models (Full, Center, Periphery, GCS) based on features of different pretrained CNN architectures: ResNet18, ResNet50, ConvNeXT. The temporal delay between the encoding of peripheral and central information persists when using features from other CNN architectures. (TIF) [file pcbi.1014371.s008.tif]

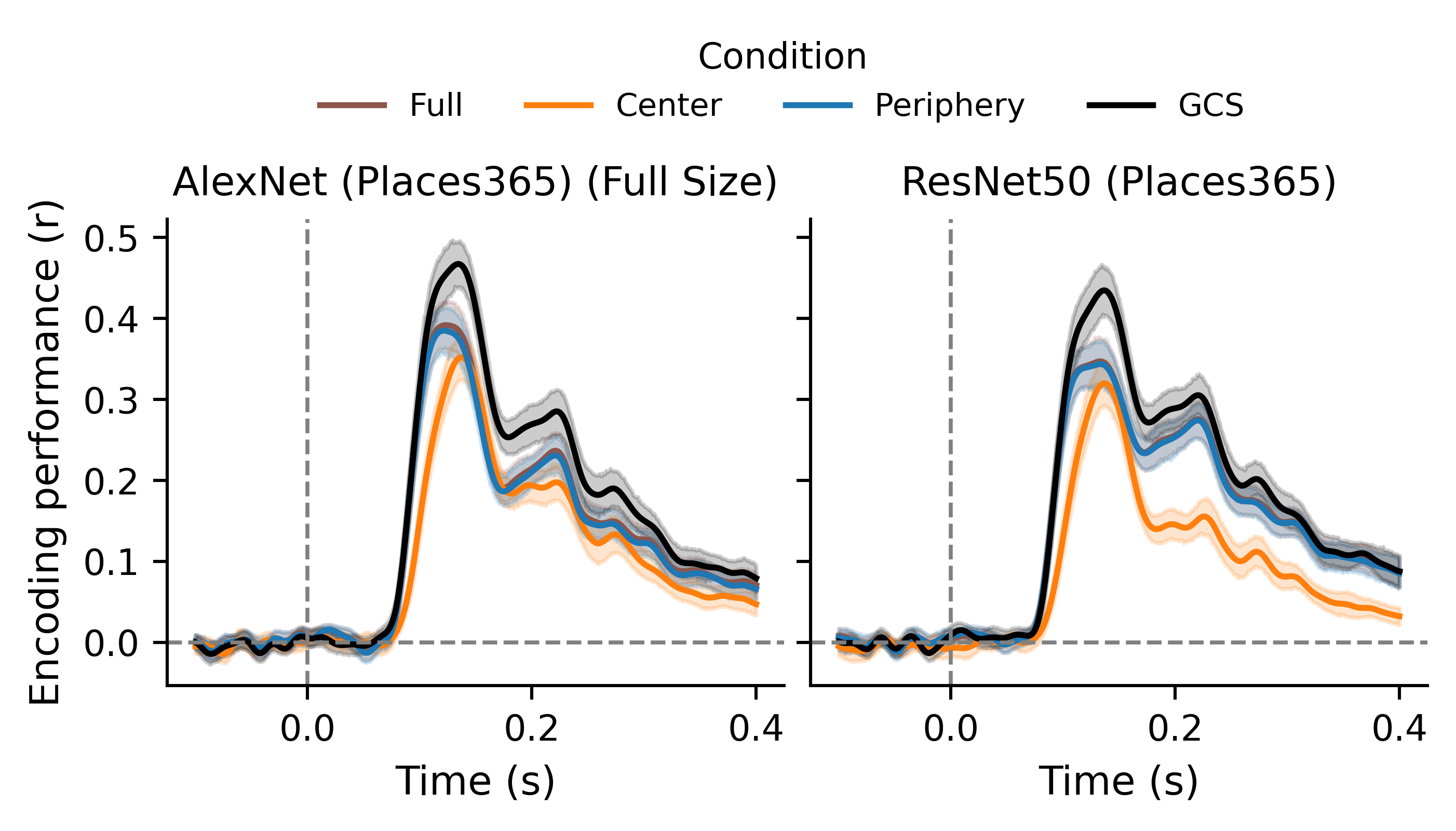

Supplement: S8 Fig — Average encoding performance (r) across participants of the four models (Full, Center, Periphery, GCS) based on features of different CNN architectures trained on datasets other than ImageNet: AlexNet and ResNet50 trained on Places365. The temporal delay between the encoding of peripheral and central information persists when using features from CNNs trained on a dataset and task (scene categorization) that is centered on scene information instead of on object information. (TIF) [file pcbi.1014371.s009.tif]

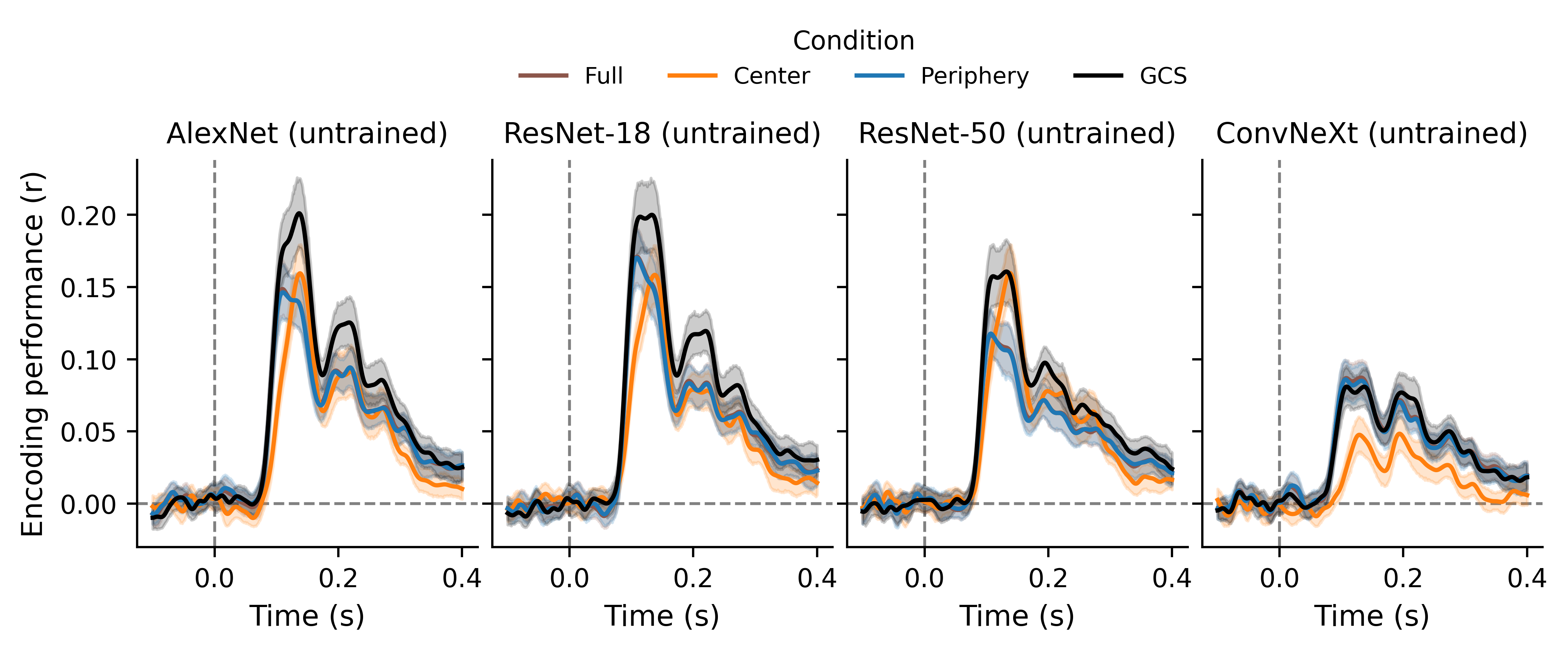

Supplement: S9 Fig — Average encoding performance (r) across participants of the four models (Full, Center, Periphery, GCS) based on features of different untrained CNN architectures. The temporal delay between the encoding of peripheral and central information persists when using features from untrained CNNs. (TIF) [file pcbi.1014371.s010.tif]

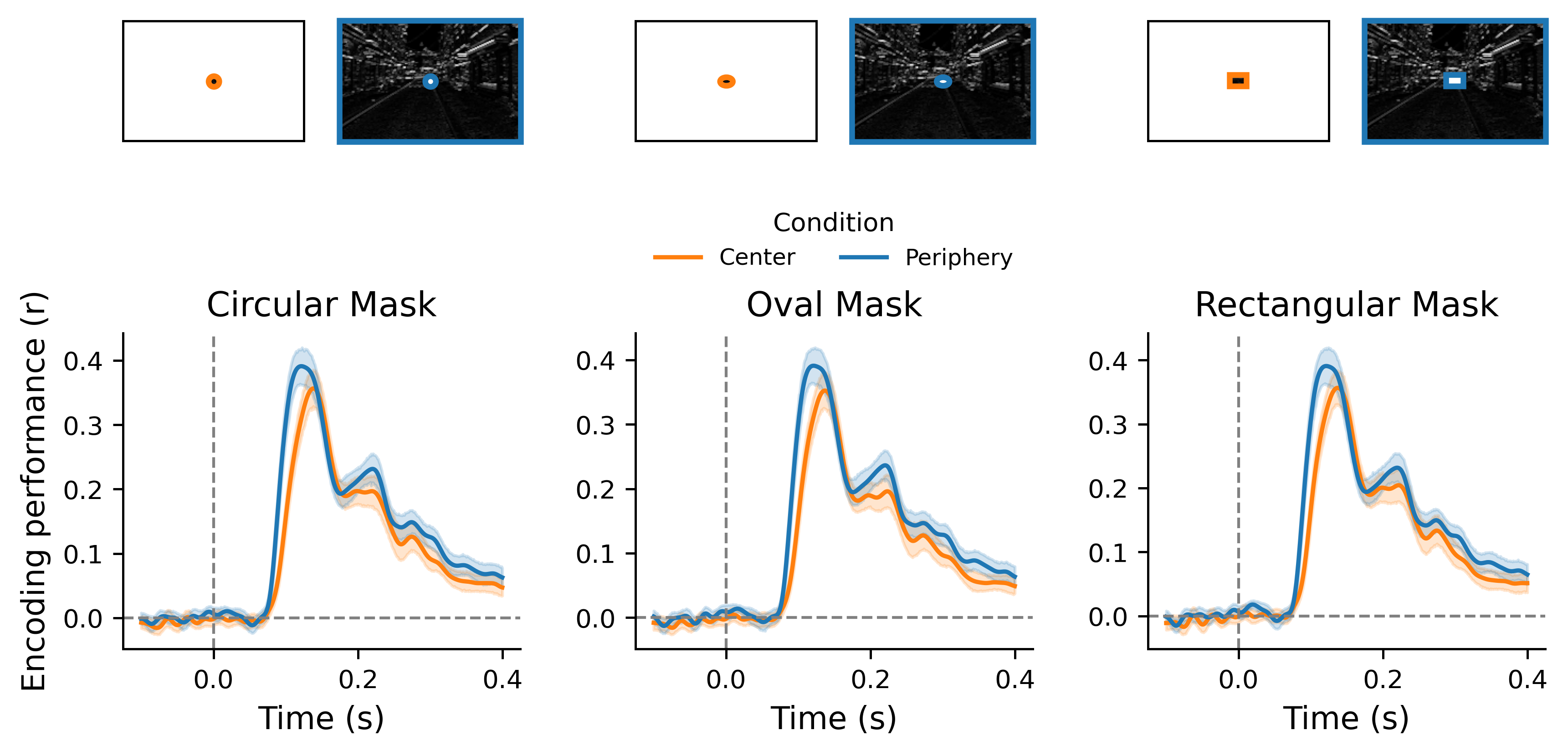

Supplement: S10 Fig — Average encoding performance (r) across participants of the Center and Periphery models using different crop types: instead of using circular crop (see Fig 2), we used oval crops or rectangular crops, both with a horizontal radius that is larger than the vertical radius. (TIF) [file pcbi.1014371.s011.tif]

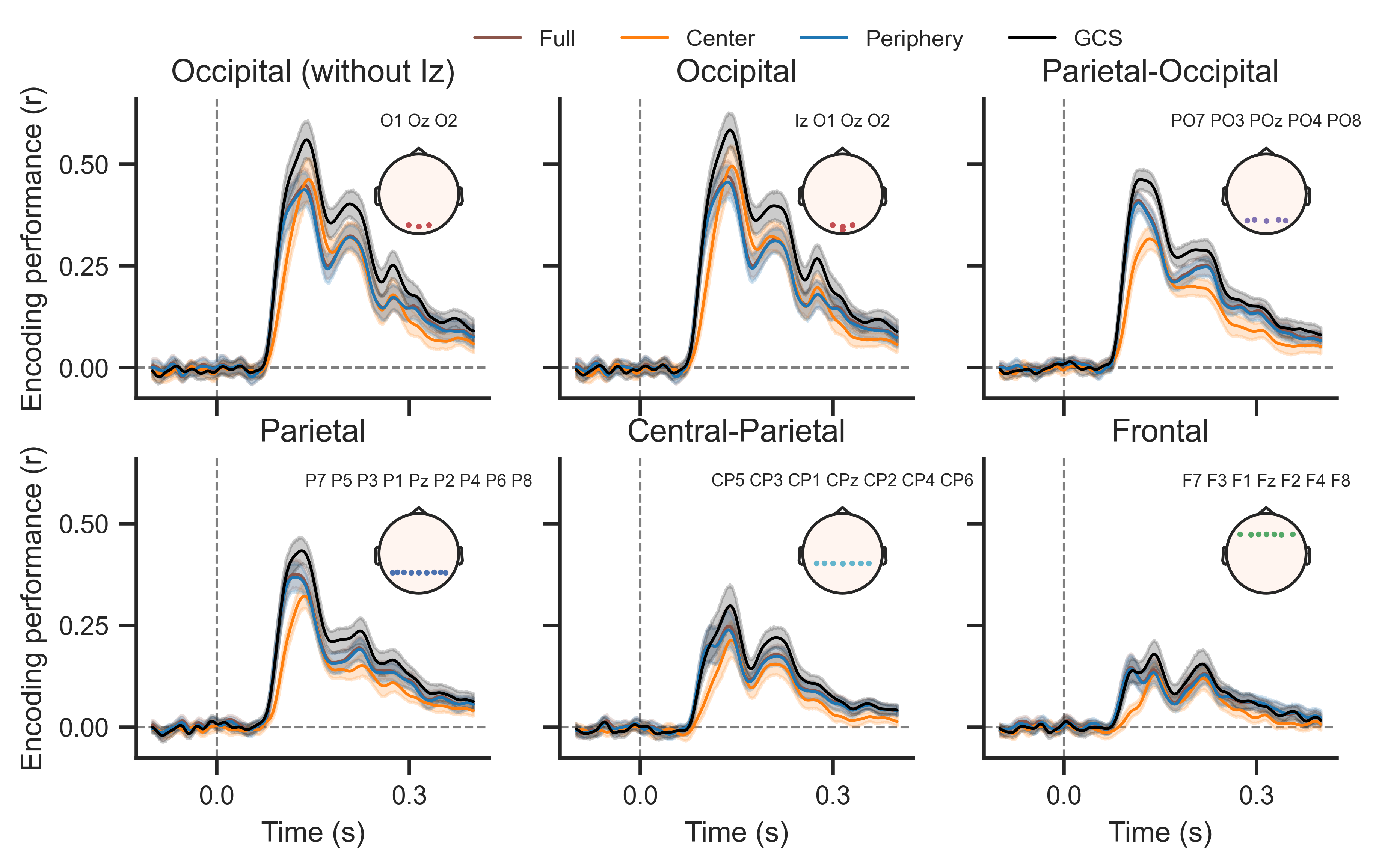

Supplement: S11 Fig — Average encoding performance (r) across participants for the four encoding models (Full, Center, Periphery, GCS) averaged across groups of electrodes. Topoplot insets indicate the location of the electrode included per group and names above follow the same order, from left to right. The temporal delay between the encoding of central and peripheral information persists after averaging the results across spatially-adjacent electrode groups. (TIF) [file pcbi.1014371.s012.tif]

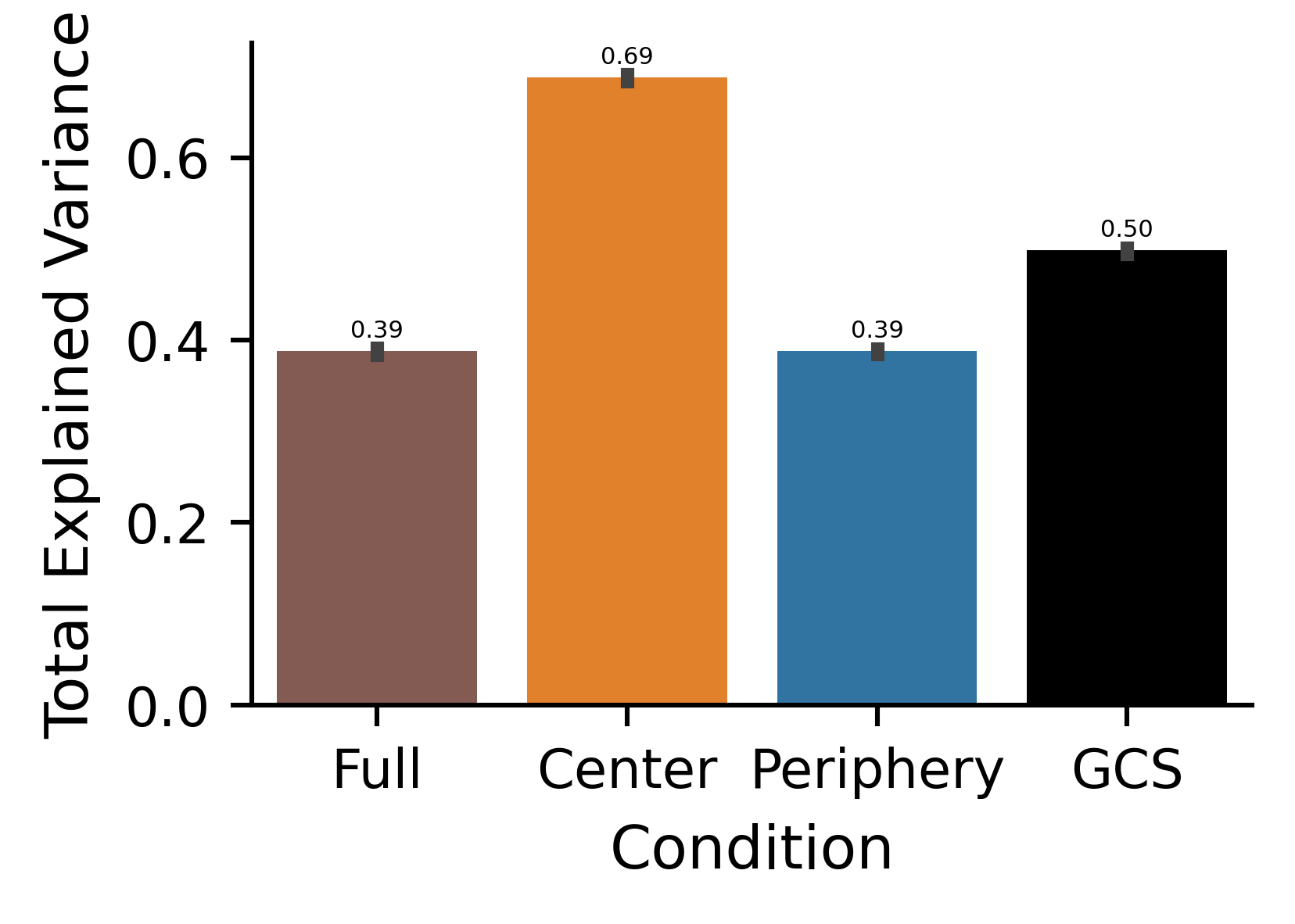

Supplement: S12 Fig — Average explained variance (summed across all 100 components) across participants for the four encoding models (Full, Center, Periphery, GCS) of the PCA fitted on the feature maps after applying the respective spatial sampling. (TIF) [file pcbi.1014371.s013.tif]
