# Supplementary material for: Linking retinal sampling in neural encoding models to temporal profiles of visual processing in humans
Source: PLoS Comput Biol. 2026 Jun 30;22(6):e1014371. doi: 10.1371/journal.pcbi.1014371 (PMC13340806; doi:10.1371/journal.pcbi.1014371)
Supplement: S1 Text — (DOCX) [file pcbi.1014371.s005.docx]

To test whether the absence of eye-tracking data led to artifacts created by eye movements (saccades), we repeated all modeling experiments (see section 3.2) for the 6 participants for whom eye-tracking data is available and separated data into trials where no saccades (defined as a fixation 1 dva away from the center of the fixation cross during the stimulus presentation) were performed and trials where saccades were performed. We repeated each analysis for each set of EEG data (fixation set, saccade set) and show the results in the Figure in S6 Figure. If eye movements were the main reason for encoding model differences between stimulus conditions, these differences should disappear for the analysis on the fixation set and remain for the analysis on the saccade set. Consistent with no influence of eye movements, we find that the temporal difference between center and periphery condition disappears when using only saccade trials, directly showing that saccades alone cannot explain the observed temporal differences (see Figure in S6 Figure).
