# Supplementary material for: Linking retinal sampling in neural encoding models to temporal profiles of visual processing in humans
Source: PLoS Comput Biol. 2026 Jun 30;22(6):e1014371. doi: 10.1371/journal.pcbi.1014371 (PMC13340806; doi:10.1371/journal.pcbi.1014371)
Supplement: Attachment — Submitted filename: Review_responses.pdf [file pcbi.1014371.s014.pdf]

Editor comments

We received three high-quality reviews. All reviewers found interest with your study. Still, R3 challenges some of the novelty claims, a point that warrants extra attention in the revision. Please also make sure that the dataset can be retrieved (R2).

> We thank the Editor and all three reviewers for their comments and suggestions to improve our manuscript. Detailed responses to each comment are found in blue font below.
>
> In addition, we have paid extra attention to revisions that highlight the novelty of our findings; detailed responses can be found to R2 point 9 and R3 point 1 below, and in edits in the manuscript in the Abstract (page 1), the Introduction (page 2, lines 49-60 and 84-88), and in the Discussion (page 15 lines 418-420, page 17 lines 486-498, page 19 lines 592-618, page 20 lines 641-656, and page 21 line 669).
>
> As explained in responses to R2 the dataset is now openly retrievable on OSF (https://osf.io/tu34h/).

Reviewer 1

In “Linking retinal sampling in neural encoding models to temporal profiles of visual processing in humans,” the authors propose different spatial sampling schemes for feature maps from CNNs used in encoding models, which reveal distinct temporal processing profiles of spatial information. Specifically, they differentially sample the fovea vs the periphery to more closely resemble biological vision. This work shows that peripheral information explains the EEG signal more effectively at early time points, while foveal information explains it more effectively at later time points. Importantly, this result demonstrates how encoding models can be leveraged to uncover signatures of retinotopic organisation in EEG signals without requiring experimental manipulation of the visual stimulation for the participants. Nonetheless, the authors also demonstrate that similar findings can be obtained with experimental manipulation (by distinct spatial visual stimulation). Finally, the authors provide a data-driven method for estimating which portions of the visual field contribute most to explaining the EEG signals of different electrodes, thereby reinforcing previous findings. Overall, this is a very well-written manuscript; the figure legends are very comprehensive, the results are nicely presented, and the methodology is self-sufficient and sound. Scientifically speaking, this work offers a well-balanced combination of computational modelling and experimental manipulation.

My only suggestion is to better contextualise these findings with other work that pinpoints differences in temporal response profiles of specific visual areas and how they relate to their known retinotopic biases. There is only a brief mention of that in lines 425-427, and this could be a nice point to explore a bit more. Some references are:
- Carrasco et al.,Speed of visual processing increases with eccentricity https://pmc.ncbi.nlm.nih.gov/articles/PMC3077107/
- Silson et al., Systematic effects of retinotopic biases and category selectivity across human occipitotemporal cortex https://www.biorxiv.org/content/10.1101/2024.11.04.621840v1.full.pdf+html
- Potentially this one: Kim et al., Characterizing Spatiotemporal Population Receptive Fields in Human Visual Cortex with fMRI https://pmc.ncbi.nlm.nih.gov/articles/PMC10866195/#jneuro-44-e0803232023F7

Response

We thank the reviewer for the positive summary and the suggestions for integrating the manuscript better with existing literature.

We appreciate the pointers to specific references on differences in temporal response profiles of specific visual areas. We now include those references in the Discussion section together with an outline of how our work contributes to integrating study of temporal dynamics with that of retinotopic specialization and organization.

Action

We have added a paragraph to the Discussion in section 2.8 (lines 641-656) on recent efforts to link retinotopy and temporal dynamics in models of visual processing.

Minor:

1) In Section 1.1, some acronyms were not previously introduced, e.g. RSVP (rapid serial visual presentation) in line 87, ERP (event-related potential) in the Figure 1 legend.

We have fixed these mistakes.

2) In line 102, the authors say "Wilcoxon signed-rank test" is used for statistical inference in Figure 2B, but in the legend, it says "paired t-test".

We have adjusted the description; the test that was used is the Wilcoxon signed-rank test.

3) Line 222: missing word in "captures almost ALL variance..."
4) Line 375: missing word in "compared TO electrodes..."
5) Font size in figures 6 and 7 is too small.

We have fixed these mistakes.

6) The first two paragraphs from section 2.1 seem unnecessary, as you don't discuss how those findings relate to the current study. Maybe you can shorten that subsection?

We thank the reviewer for the pointer and have now added two sentences at the end of section 2.1 (lines 455-460) that connect the findings of our study with the physiological properties of parvo- and magnocellular cells that are introduced in the first two paragraphs of this section, outlining that the temporal encoding profiles can be related to the different processing speeds as well as spatial densities of RGCs and parvo/magno cells.

7) Typos in line 570, "Amsterdamapproved", and line 579 ", scenestaken"

We have fixed these mistakes.

Reviewer 2

This is a nice, well-executed paper that demonstrates improved EEG decoding for the temporal dynamics of viewing natural scenes using models that include inductive biases based on the peripheral/central organization of the primate retina. Overall I strongly recommend publication with few changes other than asking the authors to address some of the conceptual issues I raise below.

We thank the reviewer for their positive evaluation overall and the theoretical and practical suggestions for improving the manuscript. Below, we respond to each suggestion individually.

1. I am unclear why AlexNet was used as the model architecture for the encoding models. AlexNet has some idiosyncratic features relative to more recent models (e.g., the way the skip connections are implemented, the fully connected layers at the top of the hierarchy, etc.; some of these factors *might* interact with the peripheral/central manipulation). Moreover, the standard for using encoding models for neural prediction is rapidly moving towards multiple architectures - e.g., Wang et al (2023) and Conwell et al (2024). Given the heavy lift of adding additional models to the anlyses, I don't expect this for publication, but it would be good to incorporate some discussion of this issue into the paper.

Response

We agree that AlexNet has a rather idiosyncratic architecture and that a more comprehensive comparison of our central manipulation across different architectures benefits the manuscript.

We have therefore repeated our encoding model analysis, comparing Full, GCS, Center, and Periphery manipulations, for three additional DNN architectures: ResNet18 and ResNet50 (He et al. 2016), and ConvNeXt (Liu et al. 2022). We observe the same qualitative pattern, namely a temporal delay of the Center encoding model compared to Full and Periphery as well as an overall superiority of the GCS model.

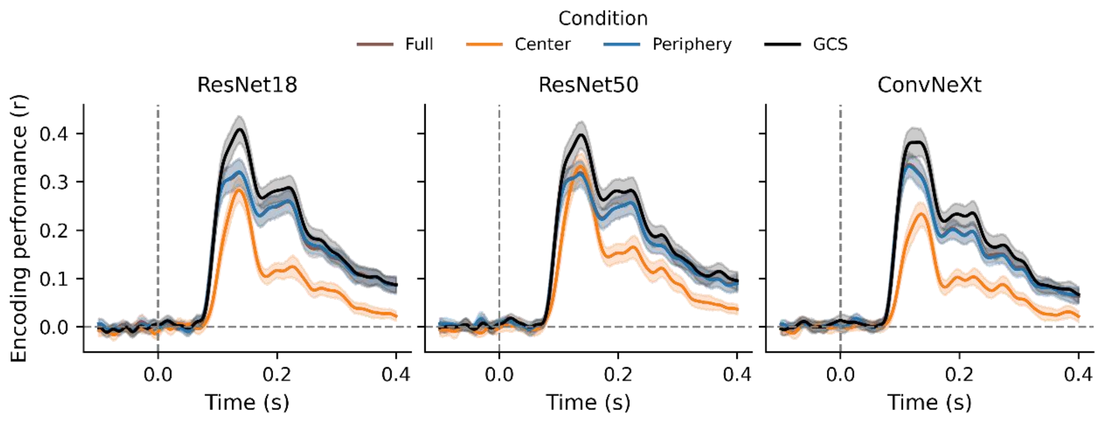

New **Supplementary Figure S8**; Average encoding performance (r) across participants of the four models (Full, Center, Periphery, GCS) based on features of different pretrained CNN architectures: ResNet18, ResNet50, ConvNeXT. The temporal delay between the encoding of peripheral and central information persists when using features from other CNN architectures.

We therefore conclude that the observed temporal differences in the EEG recordings result from modelling different spatial inputs to the encoding model and do not result from architectural idiosyncrasies. The robustness of these temporal differences across different DNN architectures further strengthens our claim that EEG recordings express temporal differences in encoding spatial information.

Action

We have included encoding model results with three alternative CNN architectures in Supplementary Fig. S8; we reference this figure in the manuscript in the Introduction in lines 72-73 and in the Results on lines 199-206.

2. Perhaps more concerning about the use of AlexNet is the fact that it is train on ImageNet images. ImageNet is a mess from the standpoint of the organization of categories (it mixes subordinate, basic, and superordinate, thereby mixing what level of visual similarity is baked into each category). ImageNet also primarily uses relatively isolated single object images - there is often little background/peripheral content. Thus, the features extracted from AlexNet pretrained on ImageNet may be somewhat idiosyncratic relative to natural scenes in terms of object extent, associated peripheral/contextual information, etc. I think the authors need to discuss this limitation/concern in some detail because it is conceivable that this choice led to some of the results obtained here. For example, is it possible that generic CNN or ViT pretrained with full-on natural scenes would show good temporal dynamic prediction without needing to introduce the retinal inductive biases that are centerpiece of this paper?

Response

We share the reviewer's concern about the flaws of ImageNet. To investigate whether the use of features from an ImageNet-pretrained neural network for the encoding models affects our findings, we have now tested additional encoding models that are based on features from DNNs trained on images from the Places365 dataset (Zhou et al. 2017), which contain more background information and are not focused on objects but rather on scene information.

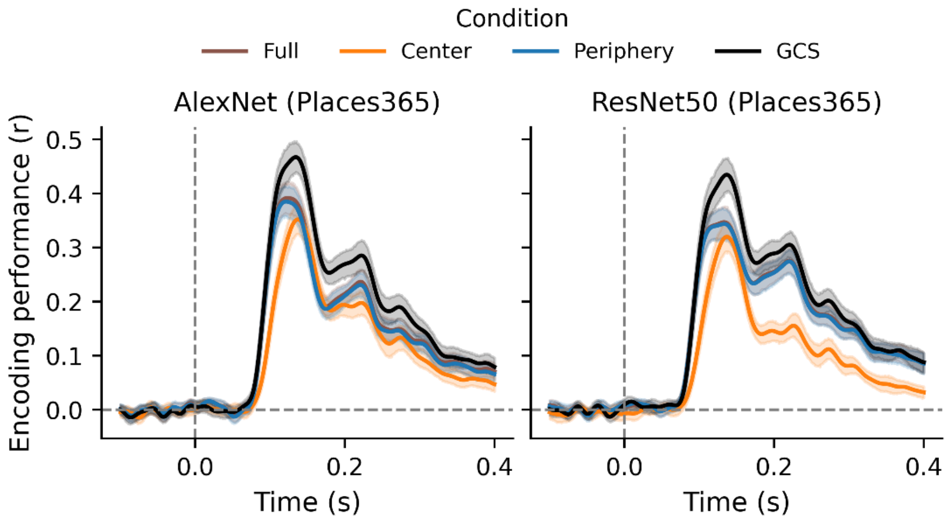

New **Supplementary Figure S9**; Average encoding performance (r) across participants of the four models (Full, Center, Periphery, GCS) based on features of different CNN architectures trained on datasets other than ImageNet: AlexNet and ResNet50 trained on Places365. The temporal delay between the encoding of peripheral and central information persists when using features from CNNs trained on a dataset and task (scene categorization) that is centered on scene information instead of on object information.

Additionally, we have tested encoding models based on features from untrained versions of the four architectures that we now include (AlexNet, ResNet18, ResNet50, ConvNeXt).

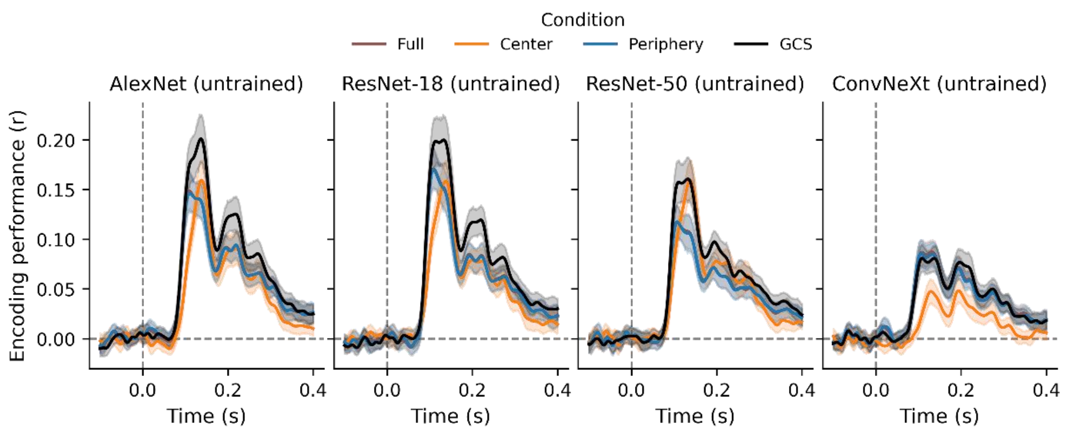

New **Supplementary Figure S10**; Average encoding performance (r) across participants of the four models (Full, Center, Periphery, GCS) based on features of different untrained CNN architectures. The temporal delay between the encoding of peripheral and central information persists when using features from untrained CNNs.

We find that for both the untrained and the Places-365-trained versions, the temporal pattern persists, suggesting that the influence of the training dataset on the results of our manipulation is minimal, and that adding retinal inductive biases is critical for observing the distinction in temporal profiles. However, as expected, we do see that the overall encoding performance decreases to about half for the untrained versions compared to the pretrained versions, equally for all encoding model conditions as well as all CNN architectures (with the exception of ConvNeXt for which the encoding performance drops even more). This confirms that the distinc temporal signatures are driven by spatial sampling, rather than feature turning.

Action

We have included results from additional, non-ImageNet trained encoding models in Supplementary Fig. S9 and S10 and now refer to these figures in the Introduction on lines 72-73 and in the Results on lines 199-206. We additionally discuss these findings in a new section 2.7 on lines 589-618.

3. Is it possible that the *low* spatial resolution of EEG actually interacts and enhances the effect of the peripheral/central manipulation? That is, if the actual signal is blurred with respect to retinotopic representations, what is the impact vis a vis the prediction accuracy of the different manipulations?

Response

Here, the reviewer is asking whether there is an interaction between the low spatial resolution of EEG and our center/periphery manipulation, and whether the observed temporal differences in encoding performance would differ when the EEG signal would be blurred. To test this, we ran an additional control analysis, whereby we first blurred the retinotopic representations of the EEG signal by averaging across all 64 electrodes

for every time point and subject, effectively removing potential effects of the spatial distribution of the electrodes on the scalp. We then repeated our encoding analysis for all four encoding models (Full, GCS, Center, Periphery) on this electrode-averaged EEG data.

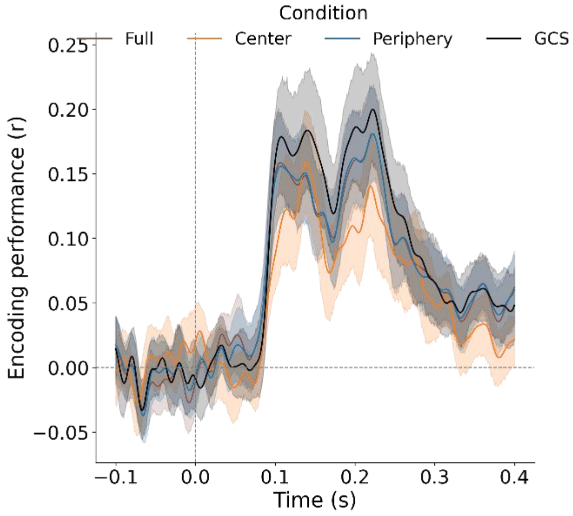

Average encoding performance (r) across participants of the four models (Full, Center, Periphery, GCS) trained and tested on EEG data that was averaged across all 16 posterior electrodes to blur retinotopic representations within the data. Even then, we observe a temporal delay in the encoding of central information.

We find that the overall encoding performance decreases for all four encoding models (likely due to an increased noise level in the data, as we now include frontal and central electrodes in the average). However, the main patterns that we point out in our manuscript are still clearly visible in this analysis, namely that (1) there is a temporal delay of the encoding profile of the Center model compared to the Periphery model, and (2) the GCS model outperforms all other models across the full time course.

This suggests that the link between the temporal dynamics and retinotopic structure of visual cortex is a global feature that is dominant in ERPs across the entire scalp.

4. Since you are address the temporal dynamics of visual processing, I was suprised that the chosen models did not also include some sort of asynchronous component at the input level - as the authors know, retinal inputs to the brain are not aligned - rather there is a asynchronous set of continous signals rate limited by the physiological refresh rate of each receptor. How might the asynchronous signal interact with and impact the model as articulated in this paper?

Response

Here, we believe the reviewer may be referring to physiological differences between magno- and parvocellular retinal cells, and specifically that they process information at different "sampling rates", which some prior works suggest can be detected in EEG responses (when using artificial stimuli, e.g., Rudvin et al., 2000).

In this work, we indeed assume that there are differences in both the spatial distribution and the temporal dynamics of magno- and parvocellular cells and the functional and structural consequences thereof. Specifically, we base our model on empirical findings of the density of retinal ganglion cells (Da Costa et al. 2022), which is tightly linked to the distribution of these cells, to distinguish between a foveal/central region and a peripheral region. From there, the aim of this work is to identify the temporal profiles of the encoding of central and peripheral information by these distinct retinal populations. Rather than incorporating explicit temporal differences in temporal integration in the model directly, we here aimed to measure the consequences on scalp EEG encoding profiles when using different spatial inputs. In other words, we here *observe* temporal differences as a result of differential spatial sampling, rather than putting these differences as hypotheses in the models themselves.

However, we believe that explicit incorporation of temporal sampling differences between different populations of retinal receptors in EEG encoding models would be an interesting avenue to explore in future work, following related lines of research on electrocorticographic recordings by Groen et al. (2022) or fMRI recordings by Kim et al. (2024).

Action

We have added a paragraph in the suggestions for future work section to the Discussion, in lines 641-656, proposing that future models integrate the modeling of both temporal dynamics and retinotopic organization for a more holistic model of visual processing.

5. Why didn't the authors adopt oval crops for inputs aligned with the shape of the retina? I wonder if there might be corner effects for these inputs that are interacting with the different models?

Response

We thank the reviewer for asking us to motivate this design choice. We chose to use circular crops because the GCS transform from Da Costa et al. (2024) is designed to be circular. We do not consider it likely that corner effects resulting from such crops would impact our claims, given that all of our spatial sampling manipulations are performed post-hoc on the same set of extracted CNN features, and the processing of the visual information by the CNN itself is thus not influenced by the idiosyncrasies of the spatial sampling. Moreover, in a previous version of this manuscript (Mueller et al. 2024, bioRXiv v1), we used rectangular crops, which (similar to oval crops) are wider along the horizontal axis compared to the vertical axis, and we found identical results. However, the suggestion of the reviewer is an excellent opportunity to further verify the robustness of our findings, and we therefore now include additional analyses for oval rather than circular crops. Direct comparison of all three types of crops (circular, oval, rectangular) shows that these yield equivalent results.

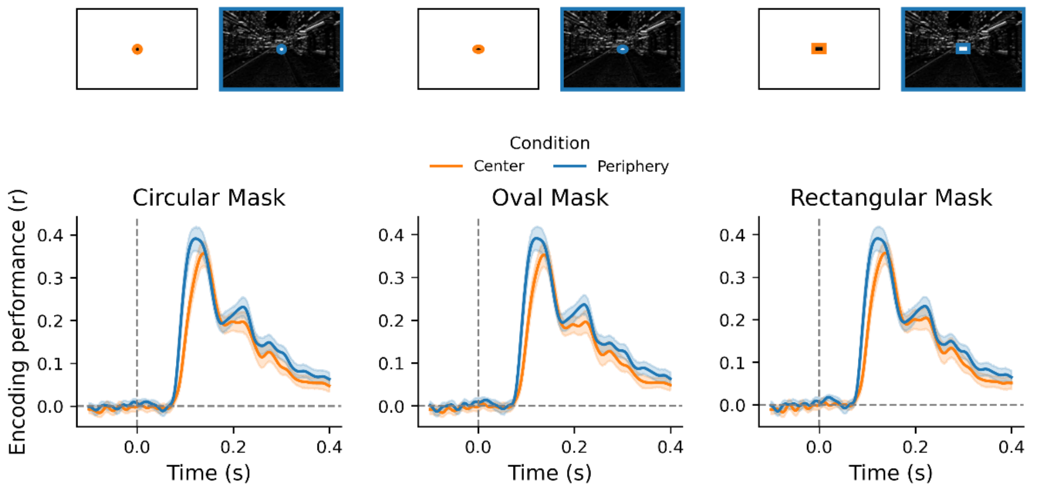

New **Supplementary Figure S11**; Average encoding performance (r) across participants of the Center and Periphery models using different crop types: instead of using circular crop (see Fig. 2), we used oval crops or rectangular crops, both with a horizontal radius that is larger than the vertical radius.

Action

We ran additional encoding models using oval crops instead of circular crops and find that the overall pattern remains similar: we observe a temporal delay of the encoding of central information compared to peripheral information.

We have included the above results in Supplementary Fig. S11 and now refer to this figure in the Results on lines 199-206.

6. The point about large-field stimulation being necessary to reveal peripheral temporal profiles

is an interesting one. It is striking that using the THINGS data revealed no improvement in encoding performance with GCS included. The control experiment with sm/med/lrg stimuli extents seems to address this, demonstrating that peripheral stimulation is the key. But I also wonder about image structure - going back to my point about ImageNet, might it also be possible that scenes with complex, natural backgrounds elicit peripheral processing profiles (even with a limited visual angle and falling only in the parafoveal region)?

Response

Here, the reviewer is wondering whether complex, natural image backgrounds might be sufficient to elicit peripheral processing profiles, even when the extent of stimulation is limited to parafoveal regions. We find converging two-fold evidence that complex backgrounds are not sufficient, however might be necessary, to elicit peripheral processing profiles.

First, despite the THINGS dataset consisting of images showing large, individual objects, they are never presented in isolation but always on (complex) backgrounds. Thus, the scenario described by the reviewer should hold for data obtained on THINGS images, which we do not find to be true.

Second, in our control experiment with sm/med/lrg stimuli, we only alter the extent of the stimulation but not the complexity of the image background. Still, we find that only in the large stimulation condition, there is an improvement of the GCS model over the Full model. If complex, natural backgrounds elicit peripheral processing profiles, then our Small condition should also show a similar improvement of the GCS over the Full model, but we don't see this.

In sum, we believe that our results suggest that the limited spatial extent of visual stimulation in THINGS-EEG, rather than the absence of complex backgrounds, is the reason for the absence of a GCS advantage in that EEG dataset.

7. I am intrigued by Section 1.3.2 and the result that they data-driven spatial weights actually *outperforms* the full-field models. Does this mean that explicitly including retinal inductive biases is not the best strategy, but rather, one should just build adaptive encoding models that learn spatiotemporal tuning profiles on a per electrode (or voxel, etc) basis? If so, what is the point of the prior experiments in the paper? I think the authors need to say more about this, discussing how these biases evolve/are learned and about how the data-driven result informs such learning, as well as how one might build more effective encoding models without introducing explicity inductive biases, but rather rules for learning such biases (I recommend "Encoding innate ability through a genomic bottleneck" by Shuvaev et al).

Response

We thank the reviewer for the opportunity to clarify the added value of the GCS and data-driven analysis in the manuscript.

On the one hand, the GCS is built on an interpretable magnification parameter (derived from empirical measurements of the distribution of retinal ganglion cells, RGC) and as such forms a hypothesis about how much the non-uniform RGC distribution influences the EEG signal. We find that the EEG signal is indeed highly influenced by the RGC

distribution and can be predicted significantly better when using the GCS transform as part of the encoding model. An additional analysis suggested by Reviewer 3 (see point 2D), further shows how valuable a parameter-based analysis can be: we replicate temporal differences in optimal magnification factor per group of electrodes and also confirm that the empirically derived magnification factor of 20 degrees used for the GCS on average leads to the higher encoding performance (see new Supplementary Fig. S6).

On the other hand, our data-driven method finds the best parameters (i.e. spatial weighting) to explain the measured, spatially-pooled, noisy EEG recordings. As such, it not only finds parameters that explain retinal transforms well but that at the same time also account for subsequent processes, such as in LGN or visual cortex, that contribute to the final EEG measurements. Therefore, contrasting the outcomes of the data-driven experiment with that of the hypothesis-driven GCS analysis allows us to infer what additional factors influence the EEG signals, beyond a foveal magnification that is grounded in the distribution of retinal ganglion cells. We find that the importance maps reveal patterns that are not modelled by the GCS: for example, for parietal electrodes at early time points (ca. 95 ms), we find that all spatial/retinal locations contribute equally to the encoding performance with non-zero contribution (approx. r=0.2; see Fig. 6B).

In sum, by using the GCS we showcase that EEG signals contain a substantial amount of information on how the visual system spatially samples the input and as such can be better predicted when considering the structure of the retina. We then go beyond modeling only the influences of retinal structure on EEG predictability towards a more complete, yet flexible way of modeling EEG recordings.

We further agree with the reviewer that a more advanced and biologically plausible version of identifying inductive biases would be through imposing learning constraints that humans are exposed to, such as energy or volume constraints on the brain.

Action

We have updated Discussion section 2.6, lines 567-588, to include a discussion of how learning constraints could be used to build models that develop human-like inductive biases as a result of these constraints.

8. I would love to hear something from the authors about how introducing these inductive biases might improve model performance in "AI" tasks (e.g., not brain prediction). Some speculation here would be welcomed/interesting. What kinds of tasks? In what way? More efficient learning? Higher performance? Etc.

Response

We thank the reviewer for the invitation to speculate about potential benefits of retinal inductive bias for AI tasks. Given that large models that require far more training data than humans to perform a single task have reached accuracy levels that go beyond that of humans, it is hard to imagine that incorporating inductive biases would further

improve model performance (if measured purely on IID accuracy). However, given the prevailing superiority of humans on noisy tasks (such as cluttered or ambiguous images, or adversarial attacks) as well as their robustness to conflicting image information (texture/shape bias), these kinds of inductive biases might lead to improvements of model robustness. Prior work has demonstrated such positive effects on robustness when using retinal-inspired foveation or blurring (e.g., Deza et al. 2020, Jang et al. 2024)

Action

We have added section 2.6 in the Discussion outlining how inductive biases might contribute to future improvements of AI, specifically for robustness to noise and learning efficiency, on lines 567-574.

9. While I like the paper and the approach, I wonder how much is really new here in terms of our understanding of the visual system and the coarse to fine dynamics of natural scene perception. The basic conclusions of this paper are – to my mind – well established as basic principles of visual processing – particularly in terms of the magno/parvo split. Are the authors providing new insights? If so, they should do a better job what is really new beyond showing that adding in the appropriate retinal inductive biases does lead to better aligned encoding models.

Response

We thank the reviewer for the invitation to highlight the novelty of our manuscript. Given the similarity with a point raised by Reviewer 3, we respectfully refer to the more extensive explanation we give to R3 (point 1), and here summarize the main aspects.

Our three main contributions are as follows:

- First, we find direct evidence for unique temporal encoding profiles for foveal and peripheral processing by applying a model-based approach on natural images, without the need to construct separate experimental conditions for foveal processing and peripheral processing.
- Second, spatial reweighting has a much stronger effect on encoding performance than model architecture or training regime (previous work has pointed out that there are only minimal differences in encoding performance between different architectures, e.g., Conwell et al. 2024; thanks to the suggestion by the reviewer, we now show a similar pattern of robustness across CNN architectures, training dataset, and even pretraining itself in our manuscript; see previous responses, Figures S8-11, and lines 592-618).
- Third, retinotopy is grounded in the spatial/topographic organization of visual cortex. Thus, given the low spatial resolution of EEG recordings, it is further assumed that retinotopic influences on EEG recordings are negligible. Here, we show that a substantial amount of retinotopic information is directly reflected in temporal profiles of human EEG recordings from natural scene viewing.

Action

To better highlight the novelty of this manuscript, we have adapted the last part of the Abstract, lines 49-60 and 84-88 in the Introduction, and lines 418-420, 592-618, 641-656, and 669 in the Discussion.

Misc comments:

First line of the abstract is a bit of an overstatment: "human visual cortex is largely defined by the retinotopic tuning of populations of neurons". While this is true for the earlier parts of the visual cortex, this is no longer the case in so-called "higher-level" of ITC. For example, there is a sparse representation that is based on visual and functional/semantic similarity in higher-order regions - FFA, PPA, PPA, OPA, EBA, Tools, Food, etc. - at this level, retinotopy is not the driving force.

We agree this statement was too strong and have now changed the opening sentence to describe retinotopy as a 'key organizing principle' rather than the 'defining' force. We do respectfully note that recent fMRI research shows that many of the higher-order visual regions you mention overlap retinotopic maps and/or show visuospatial biases, as summarized in our recent TICS review (Groen, Dekker, Knapen, Silson, 2022). However, it is currently unclear to what extent retinotopy plays a *driving* role in the organization of these regions, and it is certainly not the only organizing principle.

The Open Amsterdam Data Set (OADS) is referenced as being publicly available, however no URL is provided and a (somewhat cursory) web search does not reveal a download location (oddly the search does point to an earlier paper using the OADS that also suggests the dataset is available, but doesn't point to where).

We apologize for the difficulties in accessing the dataset. The dataset is now publicly available on OSF (https://osf.io/tu34h/).

Relatedly, the authors state "Original (non-blurred) stimuli will be made available upon request" - I much prefer, given modern standards, that the stimuli be made available along with the code on the github site.

We again apologize for the confusion around the accessibility of our dataset. A version containing images in which faces and license plates were blurred is publicly accessible on OSF (https://osf.io/tu34h/files/figshare). However, due to privacy constraints, we can only share the original (non-blurred) stimuli with other researchers on a case-by-case basis (similar to ImageNet). We have now clarified this in the Data availability statement (lines 1019-1020).

Please proof the manuscript - there were various typos, odd word choices, etc. Examples:
p. 2, li 30: combining local sampling (inspired by pRF models) OF rich feature representations (from CNNs) - I think you mean with
p. 19, li 570: "Amsterdamapproved"

We thank the reviewer for the nudge; we have fixed these mistakes and checked the revised manuscript for typos and readability.

Reviewer 3

The authors build CNN-based linearized encoding models to predict ERP amplitudes elicited by large-field, high-resolution natural scenes. They compare four ways of handling spatial sampling of CNN feature maps: using the full feature map (“Full”), keeping only a tiny central crop (“Center”), removing that same central crop (“Periphery”), and applying a retinal ganglion cell sampling (GCS) transform that magnifies central regions while down weighting periphery (“GCS”). They report (i) improved encoding performance with GCS, (ii) distinct temporal profiles for center vs periphery information, (iii) an additional EEG experiment with explicit aperture stimulation to validate center/periphery temporal differences, and (iv) a “fully data-driven” random-sampling method to recover spatial tuning maps from EEG.

This is a well-executed study that does provide some new insights into the spatiotemporal use of visual information in complex natural scenes. There are several places where the clarity of the methodology can be improved. Further, although there are several fascinating takeaways from these analyses, the core contribution (coarse to fine processing with linearized encoding models) may not carry enough novelty for this journal.

We thank the reviewer for the positive evaluation and the suggestions to improve the clarity of the methodology as well as the strength of our claims. Below, we respond to each point individually.

-1- Novelty and contribution
The core pipeline (CNN features → PCA → linear regression → correlation with neural data) is standard for encoding work, and the core result (coarse to fine processing) has been established for some decades. That said, the authors' retina-inspired sampling of CNN activation maps is a nice contribution, especially when combined with the replication experiment (though with N=10, this seems a little tacked on at the end). I leave it up to the editor to determine what novelty threshold should be applied here.

Response

We welcome the invitation to clarify the novelty of our findings. Below, we outline three concrete contributions our work provides and also specify some broader implications.

First, our work provides direct evidence for a coarse-to-fine (CTF) hierarchy in scene perception derived from distinct temporal encoding for peripheral versus central scene parts. The conceptual idea of a CTF hierarchy in visual processing goes back to, for example, the famous Navon letter task (Navon 1977), and a wide range of neural and cognitive mechanisms have been proposed to underlie CTF processing (ranging from differential processing of low- and high spatial frequencies, to spike-time ordering, up to sequential, Bayesian decision-making processes; see Hegdé, 2008, for an overview). For scene perception specifically, prior evidence for CTF processing mostly derives from behavioral rather than neurophysiological findings (e.g., Oliva & Torralba, 2006). By applying an encoding pipeline to a large-scale dataset of EEG responses, we

demonstrate temporally separable central vs. peripheral information processing in scene perception, providing neural evidence of a *specific form* of CTF based on spatial sampling.

Second, our results demonstrate this form of CTF in the neural processing of *natural stimuli* (real-world scenes) during *natural stimulation* (large-field images). Much of the evidence for CTF-like processes in human perception comes from studies using artificial stimuli – e.g., (Navon) letters, Kanizsa triangles, or grayscale face cutouts – and artificial experimental manipulations - e.g., positioning stimuli in different parts of the visual field (Bar 2004; Hegdé 2008; Neri 2011; Goffaux et al. 2011; Petras et al. 2019). Since naturalistic and artificial stimuli can yield diverging outcomes (Kayser et al. 2004; Hasson et al. 2010; Martin and Schröder 2013; Talebi and Baker Jr 2012; David et al. 2004), it is important to investigate whether findings obtained with artificial stimulation conditions generalize to more naturalistic viewing settings. Our modeling results on full-field scenes, together with the replication experiment in which we 'artificially' stimulate only the center or periphery, provide a key example of a robust finding obtained with a natural stimulus that generalizes across experimental settings. Another key observation of this additional experiment is the effect of spatial extent – many (EEG/fMRI) studies on visual perception typically use small, foveal stimuli, which our results suggest do not evoke a peripheral processing signature. This can be seen as evidence that such stimuli do not adequately engage the CTF hierarchy present in natural vision.

A third key insight from our work is that encoding models that incorporate spatial weighting (GCS and Sampling, see Fig. 7) consistently outperform those without spatial weighting, even though the latter are standardly employed in EEG encoding. Prior work has shown that considering the retinotopic organization of visual cortex benefits encoding models for fMRI responses to visual stimuli on a per-voxel basis, e.g. in the form of feature-weighted receptive field models (St-Yves et al. 2018). However, it is unclear how such voxel-based estimates should translate into EEG encoding profiles. Crucially, we show that the GCS transform captures temporal dynamics associated with both foveal and peripheral processing (and outperforms the Center, Periphery, and Full models), thus suggesting that a global image transform is a promising, simple method to model retinotopic input weighting for EEG signals. This also argues against the common notion that EEG signals do not contain information about spatial sampling in visual cortex. This insight is likely to be relevant for researchers interested in optimizing prediction performance on EEG signals, e.g. in the context of BCIs or brain reading.

On a broader note, our work adds to the growing body of evidence of the utility of DNN-based encoding models to test hypotheses about neural information processing in the brain under naturalistic stimulation conditions (more formally described in the neuroconnectionist program by Doerig et al., 2023). The pros and cons of artificial versus naturalistic stimulation in visual perception research have long been (and continue to be) debated (Movshon & Rust 2005, Felsen & Dan 2005, Zhang et al. 2021). Natural images better approximate real-world experience, but inherent covariation in their stimulus properties makes it difficult to test hypotheses about separate processing of those properties (Malcolm, Groen & Baker, 2016). However, altering natural images to test separate hypotheses would make them lose their "naturalness". DNN-based encoding models offer a solution to this conundrum, because they can be directly

applied to natural stimuli while still allowing manipulability of their inputs -- similar to how artificial stimulus manipulations are used in traditional experiments. As we show in this work, moving the ‘input manipulation’ component to the encoding model allows identifying specific neural transformations that improve prediction of neural responses collected during more naturalistic viewing settings, providing a promising roadmap for future work aiming to leverage DNN-based encoding models.

Action

To better highlight the novelty and contributions of this manuscript, we have adapted the last part of the Abstract, lines 49-60 and 84-88 in the Introduction, and lines 418-420, 486-498, 592-618, 641-656, and 669 in the Discussion.

-2- Several methods require more information to fully evaluate and/or need clarification
A. The spatial feature selection
It would be helpful to fully articulate that the spatial selection is done post hoc on CNN activation maps rather than re-deriving weights from pixel masking or some other procedure. It took me a couple of readings to get this straight.

Response

We apologize for the confusing description of how the spatial selection is performed and have now remedied this in the revised manuscript.

Action

We have adjusted Fig. 1C to more clearly show that features are first extracted from the CNN (AlexNet) and then the four spatial selections are performed post-hoc, on the same extracted features. We have adjusted the caption of Fig. 1C to mention this explicitly. Further, we now include an explicit statement at the beginning of the Results section noting that each spatial selection is performed on the same set of extracted features (see line 97).

B. Train/test split with different SNR
As I understand, the authors fit a low-SNR set (5 image repetitions) and then evaluate on the high-SNR set (10 repetitions). Can the authors please provide some rationale for this choice? Do the results still hold if they train on high-SNR and test on low? Or if the splits are equalized?

Response

Our rationale here is based on standard approaches in encoding modeling, which typically employ a large, low-SNR set for model training and a smaller, high-SNR set for modeling evaluation. Encoding models benefit from training on many images, more so than from training on images with many repetitions (see e.g., Fig. 7A in Gifford et al. 2022). However, the precision of assessing generalizability of the trained encoding model improves when increasing the number of repetitions (i.e. SNR) in the test set. These considerations lead us to use different numbers of repetitions for the many images in the low-SNR training set and the fewer images in the high-SNR test set.

To confirm the utility of this standard approach, we ran the suggested experiment (swapping the low/high SNR sets for training and testing). In the swapped version, the overall encoding performance decreases but leaves the temporal differences intact (see Figure).

We believe this is due to the fact that fitting the encoding model on a high-SNR set allows it to pick up on signals that are likely not present, or less detectable in the low-SNR set, leading to lower encoding performance overall. In other words, the model overfits on the cleaner high-SNR data and then fails to generalize to the more noisy, low-SNR data.

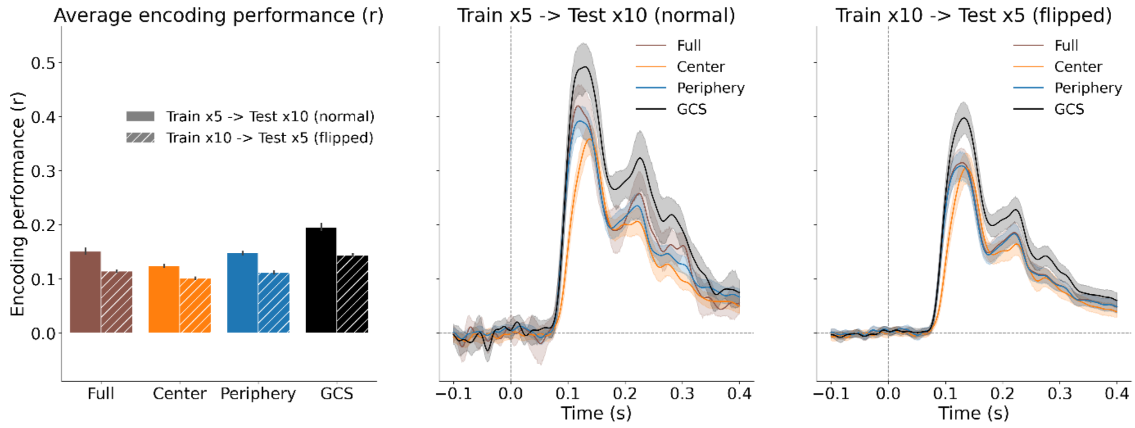

Average encoding performance (r) across participants of the Full, Center, Periphery, and GCS models, averaged across all time point after stimulus onset (left panel), as well as over time for two different cross-validation regimes (middle and right panels): training on the low-SNR (5 reps) set and testing on the high-SNR (10 reps) set, or vice versa.

C. Random sampling of pixel-wise contribution map

Some critical implementation details are missing here. How were pixels selected (i.e., from what distribution?) How many pixels per iteration? Was this stratified by eccentricity? Further, it's not clear whether the scoring correlation was computed on the same data used to fit the regression weights. If so, I would like to see a cross-validated version of this analysis to truly test generalizability.

Response

We apologize for the missing information. Pixels were selected by drawing 10 times a row and column index from a uniform distribution and then creating a 4x4 pixel patch around this location while replacing overlapping patches. Thus, in each iteration 160 pixels were selected from the first layer. For subsequent layers, the same mask was resized and reused to ensure overlapping spatial locations across layers. Pixel selection was not stratified by eccentricity.

Importantly, the scoring correlation was computed on independent test data, i.e. in a cross-validated manner, i.e. correlation values that were used to construct the contribution maps were obtained on the training set. For the subsequent assessment of the encoding performance of the spatially-optimized model, the contribution maps (obtained on the train set) were used as spatial weights to fit a new encoding model on the same train set, which was then evaluated on the test set. We then compared the resulting encoding performance on the test set against the other encoding models.

Action

We extended the description of this part of the Methods by adding more detail about how masks were created in lines 880-888 and by emphasizing in line 895 that contribution maps were obtained from sampling exclusively on the training set.

D. GCS parameterization

The authors used the default foveal size of 20 degrees. Given the paper's own suggestion that

varying magnification could matter, it would be nice to vary this parameter to strengthen the claim.

Response

We thank the reviewer for this insightful suggestion. We share the reviewer's intuition that the GCS parameterization is important to investigate. We therefore repeated our encoding analysis with a range of different GCS magnification factors and compared these results across time as well as across different electrode ROIs.

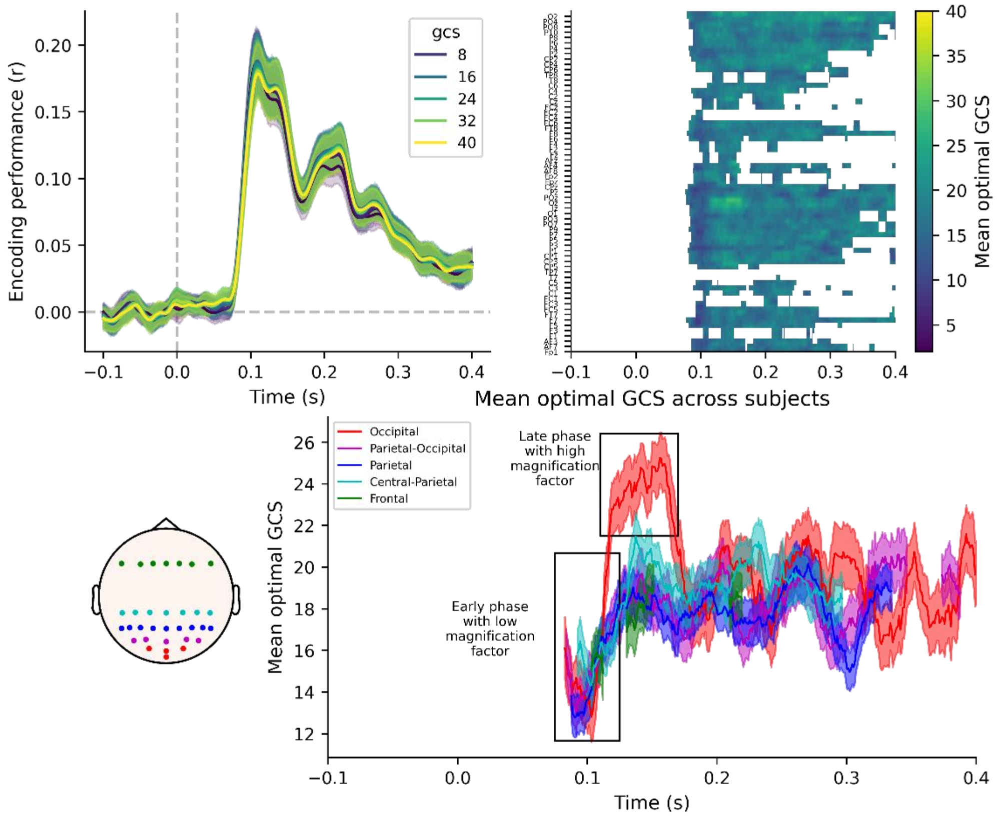

New **Supplementary Figure S7**; We repeated the encoding analysis using the GCS model multiple times while varying the magnification factor. **a)** Average encoding performance (r) across participants for the GCS model using different magnification factors. **b)** Average magnification factor across participants yielding the highest encoding performance per time point, per electrode. Time points and electrodes for which the encoding performances across subjects were not significantly above 0 were removed (blank entries). **c)** Same data as in b), but averaged across groups of electrodes (colored topoplot on the left indicates the location of electrode groups)

Specifically, we examined which GCS magnification factor (of the following selection, chosen to cover a wide range of possible magnification factors: 2, 5, 7, 10, 12, 15, 18, 20, 22, 25, 28, 30, 32, 35, 40) leads to the highest encoding performance, per electrode, time point, and subject. At early time points (80-120 ms) smaller magnification factors (as low as 14) lead to the highest encoding performance across all electrode ROIs (occipital, parietal, frontal) while later in time (120-180 ms), larger magnification factors (20 and larger) lead to the highest encoding performance. Importantly, a difference between electrode ROIs emerges for later time points, whereby occipital electrodes are best explained by higher magnification factors (around 25) compared to parietal and

frontal ROIs (around 20). Strikingly, for time points later than 180 ms, all electrode ROIs seem to converge to an optimal magnification factor around 20, consistent with the default parameter suggested by da Costa et al. (2024) based on empirical measurements of the density of human retinal ganglion cells. These findings are consistent with our original results: they suggest that the weighting of foveal and peripheral information in visual cortex is not fully dictated by the physiological density of retinal ganglion cells but changes dynamically over time, following a coarse-to-fine processing hierarchy.

Action

We have now included a Supplementary Figure (Fig. S7), showing the results for varying the magnification factor of the GCS transform. We show that (1) the encoding performance varies significantly across different magnification factors, and (2) that this variation is informative about the link between retinal sampling (i.e. spatial reweighting) and the temporal dynamics of visual processing. We additionally reference this figure in the Results on lines 187-192 as well as in the Discussion on lines 537-544.

-3- "Representative" results

A good chunk of the paper's figures use Iz and Pz as "representative electrodes." While I'm keenly sympathetic to the need to pare down such high-dimensional data, this also forces the reader to trust that these electrodes are in fact representative. A more standard approach might replace the single electrode traces with averaged ROIs that would represent posterior and central electrode clusters. Further, Iz is not a standard electrode in many montages, and in some participants, can be contaminated with artifacts from the neck muscles. Using ROI averages might even make the result even cleaner.

Response

We thank the reviewer for the suggestion to present results for electrode ROIs instead of individual electrodes.

We have created additional plots showing the average encoding performance for all four encoding models for different electrode ROIs. As predicted by the reviewer, we find that the temporal differences between Center and Peripheral models for the different ROIs are highly similar to those described for the single electrodes in the original manuscript. This shows that the chosen electrodes are indeed representative, and that a potential contamination from the neck muscles does not seem to cause the observed temporal differences (see specifically the first two panels, excluding and including electrode Iz).

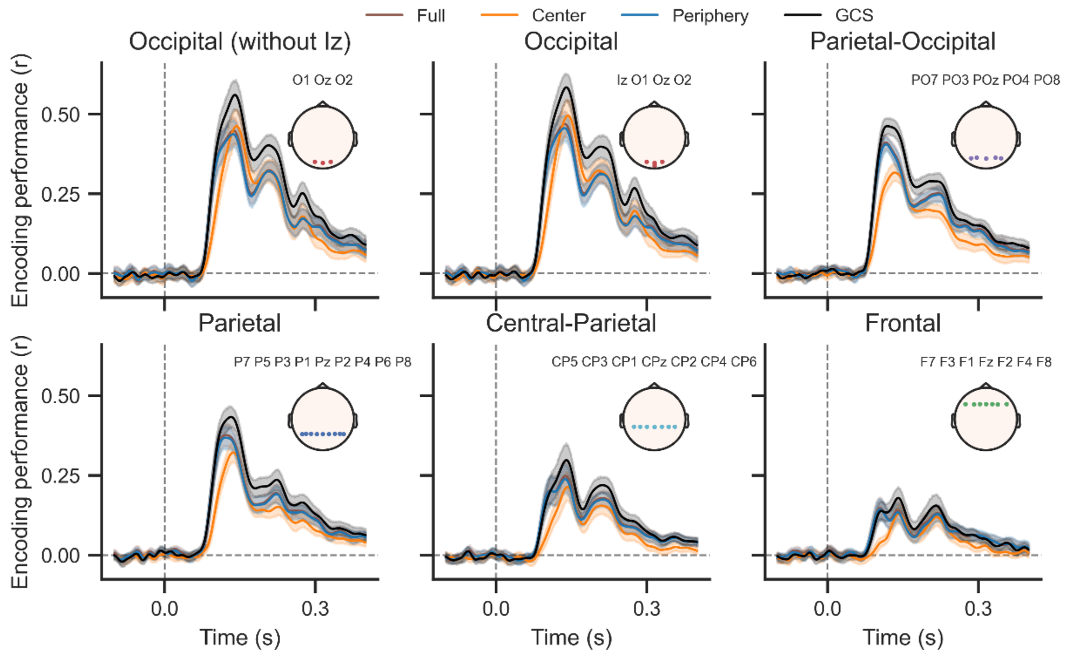

New **Supplementary Figure S12**; Average encoding performance (r) across participants for the four encoding models (Full, Center, Periphery, GCS) averaged across groups of electrodes. Topoplot insets indicate the location of the electrode included per group and names above follow the same order, from left to right. The temporal delay between the encoding of central and peripheral information persists after averaging the results across spatially-adjacent electrode groups.

Action

We have included a Supplementary figure (Fig. S12) showing these results and now refer to this Figure in the Results on lines 143-144.

Smaller issues:
Figure 1 caption: GCS? Acronym should be specified. This is also true of Line 93 in the text.

Thank you for pointing this out, we have fixed this in the revised manuscript.

Line 117: How were these timepoints chosen? I can see that at least 500 ms of post-stimulus information was recorded, so I'm curious why no later timepoints were considered. My initial thought was to avoid offset ERPs, given the RSVP presentation, but stimuli were on screen for 100 ms, so the last timepoint would also include an offset ERP.

The time points for the topoplots in these figures were chosen post hoc to illustrate the most salient differences in scalp distributions temporal profiles for the different encoding models. While there are also differences in encoding performance after these ~150 ms, we focus on earlier points because we are interested in how feedforward retinotopic sampling of information is reflected in the EEG; time points beyond 150 ms will additionally be influenced by recurrent/feedback processing (Scholte et al. 2008). We note that the t-values in D/E are corrected for comparison across all time points.

Line 713: Since the first 100 PCs were used regardless of spatial selection process, some selections might have more retained information than others in these 100 PCs. Please report the variability retained across all manipulations.

We have now included a comparison of the retained variance in Supplementary Fig. S13 (and reference this Figure in the Methods on lines 818-819), confirming that indeed some spatial selections (Center and GCS) retain more variance compared to others (Full, Periphery).

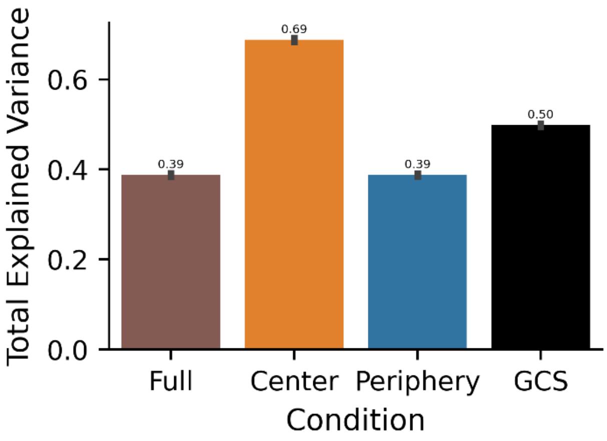

New **Supplementary Figure S13**; Average explained variance (summed across all 100 components) across participants for the four encoding models (Full, Center, Periphery, GCS) of the PCA fitted on the feature maps after applying the respective spatial sampling.

These differences in retained variance can be explained by the amount of information that got removed by the individual spatial selections, e.g. the center crop removes more pixels, making it easier for the PCA to retain more variance. However, we are interested in the effect that these specific spatial selection methods have on the encoding performance, which is primarily caused by the reduction of information (cropping the Center or Periphery, or increasing central information and reducing peripheral

information for the GCS). Thus, to fairly compare these spatial selection models, we chose a fixed number of PCA components.

Line 570: missing space
Line 633: missing space
Line 706: principal, not principle

We have fixed these mistakes.

References

Bar, M. (2004). Visual objects in context. *Nature Reviews Neuroscience*, *5*(8), 617-629.

Conwell, C., Prince, J. S., Kay, K. N., Alvarez, G. A., & Konkle, T. (2024). A large-scale examination of inductive biases shaping high-level visual representation in brains and machines. *Nature communications*, *15*(1), 9383.

Da Costa, D., Kornemann, L., Goebel, R., & Senden, M. (2024). Convolutional neural networks develop major organizational principles of early visual cortex when enhanced with retinal sampling. *Scientific Reports*, *14*(1), 8980.

David, S. V., Vinje, W. E., & Gallant, J. L. (2004). Natural stimulus statistics alter the receptive field structure of v1 neurons. *Journal of Neuroscience*, *24*(31), 6991-7006.

Deza, A., & Konkle, T. (2020). Emergent properties of foveated perceptual systems. *arXiv preprint arXiv:2006.07991*.

Doerig, A., Sommers, R. P., Seeliger, K., Richards, B., Ismael, J., Lindsay, G. W., ... & Kietzmann, T. C. (2023). The neuroconnectionist research programme. *Nature Reviews Neuroscience*, *24*(7), 431-450.

Felsen, G., & Dan, Y. (2005). A natural approach to studying vision. *Nature neuroscience*, *8*(12), 1643-1646.

Gifford, A. T., Dwivedi, K., Roig, G., & Cichy, R. M. (2022). A large and rich EEG dataset for modeling human visual object recognition. *NeuroImage*, *264*, 119754.

Goffaux, V., Peters, J., Haubrechts, J., Schiltz, C., Jansma, B., & Goebel, R. (2011). From coarse to fine? Spatial and temporal dynamics of cortical face processing. *Cerebral Cortex*, *21*(2), 467-476.

Groen, I. I., Dekker, T. M., Knapen, T., & Silson, E. H. (2022). Visuospatial coding as ubiquitous scaffolding for human cognition. *Trends in Cognitive Sciences*, *26*(1), 81-96.

Hasson, U., Malach, R., & Heeger, D. J. (2010). Reliability of cortical activity during natural stimulation. *Trends in cognitive sciences*, *14*(1), 40-48.

He, K., Zhang, X., Ren, S., & Sun, J. (2016). Deep residual learning for image recognition. In *Proceedings of the IEEE conference on computer vision and pattern recognition* (pp. 770-778).

Hegdé, J. (2008). Time course of visual perception: coarse-to-fine processing and beyond. *Progress in neurobiology*, *84*(4), 405-439.

Jang, H., & Tong, F. (2024). Improved modeling of human vision by incorporating robustness to blur in convolutional neural networks. *Nature Communications*, *15*(1), 1989.

Kayser, C., Körding, K. P., & König, P. (2004). Processing of complex stimuli and natural scenes in the visual cortex. *Current opinion in neurobiology*, *14*(4), 468-473.

Kim, I., Kupers, E. R., Lerma-Usabiaga, G., & Grill-Spector, K. (2024). Characterizing spatiotemporal population receptive fields in human visual cortex with fMRI. *Journal of Neuroscience*, *44*(2).

Liu, Z., Mao, H., Wu, C. Y., Feichtenhofer, C., Darrell, T., & Xie, S. (2022). A convnet for the 2020s. In *Proceedings of the IEEE/CVF conference on computer vision and pattern recognition* (pp. 11976-11986).

Martin, K. A., & Schröder, S. (2013). Functional heterogeneity in neighboring neurons of cat primary visual cortex in response to both artificial and natural stimuli. *Journal of Neuroscience*, *33*(17), 7325-7344.

Müller, N., Scholte, H. S., & Groen, I. I. (2024). Spatial sampling of deep neural network features improves encoding models of foveal and peripheral visual processing in humans. *bioRxiv*, 2024-08.

Navon, D. (1977). Forest before trees: The precedence of global features in visual perception. *Cognitive psychology*, *9*(3), 353-383.

Neri, P. (2011). Coarse to fine dynamics of monocular and binocular processing in human pattern vision. *Proceedings of the National Academy of Sciences*, *108*(26), 10726-10731.

Oliva, A., & Torralba, A. (2006). Building the gist of a scene: The role of global image features in recognition. *Progress in brain research*, *155*, 23-36.

Petras, K., Ten Oever, S., Jacobs, C., & Goffaux, V. (2019). Coarse-to-fine information integration in human vision. *NeuroImage*, *186*, 103-112.

Rudvin, I., Valberg, A., & Kilavik, B. E. (2000). Visual evoked potentials and magnocellular and parvocellular segregation. *Visual neuroscience*, *17*(4), 579-590.

Rust, N. C., & Movshon, J. A. (2005). In praise of artifice. *Nature neuroscience*, *8*(12), 1647-1650.

Scholte, H. S., Jolij, J., Fahrenfort, J. J., & Lamme, V. A. (2008). Feedforward and recurrent processing in scene segmentation: electroencephalography and functional magnetic resonance imaging. *Journal of cognitive neuroscience*, *20*(11), 2097-2109.

Silson, E. H., Groen, I. I., & Baker, C. I. (2024). Systematic effects of retinotopic biases and category selectivity across human occipitotemporal cortex. *bioRxiv*, 2024-11.

St-Yves, G., & Naselaris, T. (2018). The feature-weighted receptive field: an interpretable encoding model for complex feature spaces. *NeuroImage*, *180*, 188-202.

Talebi, V., & Baker, C. L. (2012). Natural versus synthetic stimuli for estimating receptive field models: a comparison of predictive robustness. *Journal of neuroscience*, *32*(5), 1560-1576.

Zhang, Y., Kim, J. H., Brang, D., & Liu, Z. (2021). Naturalistic stimuli: A paradigm for multiscale functional characterization of the human brain. *Current opinion in biomedical engineering*, *19*, 100298.

Zhou, B., Lapedriza, A., Khosla, A., Oliva, A., & Torralba, A. (2017). Places: A 10 million image database for scene recognition. *IEEE transactions on pattern analysis and machine intelligence*, *40*(6), 1452-1464.
