# Supplementary material for: Linking retinal sampling in neural encoding models to temporal profiles of visual processing in humans
Source: PLoS Comput Biol. 2026 Jun 30;22(6):e1014371. doi: 10.1371/journal.pcbi.1014371 (PMC13340806; doi:10.1371/journal.pcbi.1014371)
Supplement: Peer review history

# Peer review: Decision Letter 0

**DOI:** 10.1371/journal.pcbi.1014371.r001

**Authors:** Marieke Karlijn van Vugt, Christoph Strauch

19 Jan 2026

PCOMPBIOL-D-25-02062

Linking retinal sampling in neural encoding models to temporal profiles of visual processing in humans

PLOS Computational Biology

Dear Dr. Müller,

Thank you for submitting your manuscript to PLOS Computational Biology. After careful consideration, we feel that it has merit but does not fully meet PLOS Computational Biology's publication criteria as it currently stands. Therefore, we invite you to submit a revised version of the manuscript that addresses the points raised during the review process.

Please submit your revised manuscript by Mar 21 2026 11:59PM. If you will need more time than this to complete your revisions, please reply to this message or contact the journal office at ploscompbiol@plos.org. When you're ready to submit your revision, log on to https://www.editorialmanager.com/pcompbiol/ and select the 'Submissions Needing Revision' folder to locate your manuscript file.

Please include the following items when submitting your revised manuscript:

* A letter that responds to each point raised by the editor and reviewer(s). You should upload this letter as a separate file labeled 'Response to Reviewers'. This file does not need to include responses to formatting updates and technical items listed in the 'Journal Requirements' section below.

* A marked-up copy of your manuscript that highlights changes made to the original version. You should upload this as a separate file labeled 'Revised Manuscript with Track Changes'.

* An unmarked version of your revised paper without tracked changes. You should upload this as a separate file labeled 'Manuscript'.

If you would like to make changes to your financial disclosure, competing interests statement, or data availability statement, please make these updates within the submission form at the time of resubmission. Guidelines for resubmitting your figure files are available below the reviewer comments at the end of this letter

We look forward to receiving your revised manuscript.

Kind regards,

Christoph Strauch

Academic Editor

PLOS Computational Biology

Marieke van Vugt

Section Editor

PLOS Computational Biology

**Additional Editor Comments:**

We received three high-quality reviews. All reviewers found interest with your study. Still, R3 challenges some of the novelty claims, a point that warrants extra attention in the revision. Please also make sure that the dataset can be retrieved (R2).

**Journal Requirements:**

1) Please ensure that the CRediT author contributions listed for every co-author are completed accurately and in full.

At this stage, the following Authors/Authors require contributions: Niklas Müller. Please ensure that the full contributions of each author are acknowledged in the "Add/Edit/Remove Authors" section of our submission form.

The list of CRediT author contributions may be found here: https://journals.plos.org/ploscompbiol/s/authorship#loc-author-contributions

2) We ask that a manuscript source file is provided at Revision. Please upload your manuscript file as a .doc, .docx, .rtf or .tex. If you are providing a .tex file, please upload it under the item type u2018LaTeX Source Fileu2019 and leave your .pdf version as the item type u2018Manuscriptu2019.

3) Please upload all main figures as separate Figure files in .tif or .eps format. For more information about how to convert and format your figure files please see our guidelines:

https://journals.plos.org/ploscompbiol/s/figures

4) Some material included in your submission may be copyrighted. According to PLOSu2019s copyright policy, authors who use figures or other material (e.g., graphics, clipart, maps) from another author or copyright holder must demonstrate or obtain permission to publish this material under the Creative Commons Attribution 4.0 International (CC BY 4.0) License used by PLOS journals. Please closely review the details of PLOSu2019s copyright requirements here: PLOS Licenses and Copyright. If you need to request permissions from a copyright holder, you may use PLOS's Copyright Content Permission form.

Please respond directly to this email and provide any known details concerning your material's license terms and permissions required for reuse, even if you have not yet obtained copyright permissions or are unsure of your material's copyright compatibility. Once you have responded and addressed all other outstanding technical requirements, you may resubmit your manuscript within Editorial Manager.

Potential Copyright Issues:

- Please confirm (a) that you are the photographer of Figures 1A, 4A, 5A, S3A, and S4A, or (b) provide written permission from the photographer to publish the photo(s) under our CC BY 4.0 license.

**Reviewers' comments:**

Reviewer's Responses to Questions

**Comments to the Authors:**

**Please note here if the review is uploaded as an attachment.**

Reviewer #1: In “Linking retinal sampling in neural encoding models to temporal profiles of visual processing in humans,” the authors propose different spatial sampling schemes for feature maps from CNNs used in encoding models, which reveal distinct temporal processing profiles of spatial information. Specifically, they differentially sample the fovea vs the periphery to more closely resemble biological vision. This work shows that peripheral information explains the EEG signal more effectively at early time points, while foveal information explains it more effectively at later time points. Importantly, this result demonstrates how encoding models can be leveraged to uncover signatures of retinotopic organisation in EEG signals without requiring experimental manipulation of the visual stimulation for the participants. Nonetheless, the authors also demonstrate that similar findings can be obtained with experimental manipulation (by distinct spatial visual stimulation). Finally, the authors provide a data-driven method for estimating which portions of the visual field contribute most to explaining the EEG signals of different electrodes, thereby reinforcing previous findings. Overall, this is a very well-written manuscript; the figure legends are very comprehensive, the results are nicely presented, and the methodology is self-sufficient and sound. Scientifically speaking, this work offers a well-balanced combination of computational modelling and experimental manipulation.

My only suggestion is to better contextualise these findings with other work that pinpoints differences in temporal response profiles of specific visual areas and how they relate to their known retinotopic biases. There is only a brief mention of that in lines 425-427, and this could be a nice point to explore a bit more. Some references are:

- Carrasco et al.,Speed of visual processing increases with eccentricity https://pmc.ncbi.nlm.nih.gov/articles/PMC3077107/

- Silson et al., Systematic effects of retinotopic biases and category selectivity across human occipitotemporal cortex https://www.biorxiv.org/content/10.1101/2024.11.04.621840v1.full.pdf+html

- Potentially this one: Kim et al., Characterizing Spatiotemporal Population Receptive Fields in Human Visual Cortex with fMRI https://pmc.ncbi.nlm.nih.gov/articles/PMC10866195/#jneuro-44-e0803232023F7

Minor:

1) In Section 1.1, some acronyms were not previously introduced, e.g. RSVP (rapid serial visual presentation) in line 87, ERP (event-related potential) in the Figure 1 legend.

2) In line 102, the authors say “Wilcoxon signed-rank test” is used for statistical inference in Figure 2B, but in the legend, it says “paired t-test“.

3) Line 222: missing word in “captures almost ALL variance…“

4) Line 375: missing word in “compared TO electrodes…“

5) Font size in figures 6 and 7 is too small.

6) The first two paragraphs from section 2.1 seem unnecessary, as you don’t discuss how those findings relate to the current study. Maybe you can shorten that subsection?

7) Typos in line 570, “Amsterdamapproved”, and line 579 “, scenestaken”

Reviewer #2: This is a nice, well-executed paper that demonstrates improved EEG decoding for the temporal dynamics of viewing natural scenes using models that include inductive biases based on the peripheral/central organization of the primate retina. Overall I strongly recommend publication with few changes other than asking the authors to address some of the conceptual issues I raise below.

1. I am unclear why AlexNet was used as the model architecture for the encoding models. AlexNet has some idiosyncratic features relative to more recent models (e.g., the way the skip connections are implemented, the fully connected layers at the top of the hierarchy, etc.; some of these factors *might* interact with the peripheral/central manipulation). Moreover, the standard for using encoding models for neural prediction is rapidly moving towards multiple architectures - e.g., Wang et al (2023) and Conwell et al (2024). Given the heavy lift of adding additional models to the anlyses, I don't expect this for publication, but it would be good to incorporate some discussion of this issue into the paper.

2. Perhaps more concerning about the use of AlexNet is the fact that it is train on ImageNet images. ImageNet is a mess from the standpoint of the organization of categories (it mixes subordinate, basic, and superordinate, thereby mixing what level of visual similarity is baked into each category). ImageNet also primarily uses relatively isolated single object images - there is often little background/peripheral content. Thus, the features extracted from AlexNet pretrained on ImageNet may be somewhat idiosyncratic relative to natural scenes in terms of object extent, associated peripheral/contextual information, etc. I think the authors need to discuss this limitation/concern in some detail because it is conceivable that this choice led to some of the results obtained here. For example, is it possible that generic CNN or ViT pretrained with full-on natural scenes would show good temporal dynamic prediction without needing to introduce the retinal inductive biases that are centerpiece of this paper?

3. Is it possible that the *low* spatial resolution of EEG actually interacts and enhances the effect of the peripheral/central manipulation? That is, if the actual signal is blurred with respect to retinotopic representations, what is the impact vis a vis the prediction accuracy of the different manipulations?

4. Since you are address the temporal dynamics of visual processing, I was suprised that the chosen models did not also include some sort of asynchronous component at the input level - as the authors know, retinal inputs to the brain are not aligned - rather there is a asynchronous set of continous signals rate limited by the physiological refresh rate of each receptor. How might the asynchronous signal interact with and impact the model as articulated in this paper?

5. Why didn't the authors adopt oval crops for inputs aligned with the shape of the retina? I wonder if there might be corner effects for these inputs that are interacting with the different models?

6. The point about large-field stimulation being necessary to reveal peripheral temporal profiles is an interesting one. It is striking that using the THINGS data revealed no improvement in encoding performance with GCS included. The control experiment with sm/med/lrg stimuli extents seems to address this, demonstrating that peripheral stimulation is the key. But I also wonder about image structure - going back to my point about ImageNet, might it also be possible that scenes with complex, natural backgrounds elicit peripheral processing profiles (even with a limited visual angle and falling only in the parafoveal region)?

7. I am intrigued by Section 1.3.2 and the result that they data-driven spatial weights actually *outperforms* the full-field models. Does this mean that explicitly including retinal inductive biases is not the best strategy, but rather, one should just build adaptive encoding models that learn spatiotemporal tuning profiles on a per electrode (or voxel, etc) basis? If so, what is the point of the prior experiments in the paper? I think the authors need to say more about this, discussing how these biases evolve/are learned and about how the data-driven result informs such learning, as well as how one might build more effective encoding models without introducing explicity inductive biases, but rather rules for learning such biases (I recommend "Encoding innate ability through a genomic bottleneck" by Shuvaev et al).

8. I would love to hear something from the authors about how introducing these inductive biases might improve model performance in "AI" tasks (e.g., not brain prediction). Some speculation here would be welcomed/interesting. What kinds of tasks? In what way? More efficient learning? Higher performance? Etc.

9. While I like the paper and the approach, I wonder how much is really new here in terms of our understanding of the visual system and the coarse to fine dynamics of natural scene perception. The basic conclusions of this paper are - to my mind - well established as basic principles of visual processing - particularly in terms of the magno/parvo split. Are the authors providing new insights? If so, they should do a better job what is really new beyond showing that adding in the appropriate retinal inductive biases does lead to better aligned encoding models.

Misc comments:

First line of the abstract is a bit of an overstatment: "human visual cortex is largely defined by the retinotopic tuning of populations of neurons". While this is true for the earlier parts of the visual cortex, this is no longer the case in so-called "higher-level" of ITC. For example, there is a sparse representation that is based on visual and functional/semantic similarity in higher-order regions - FFA, PPA, PPA, OPA, EBA, Tools, Food, etc. - at this level, retinotopy is not the driving force.

The Open Amsterdam Data Set (OADS) is referenced as being publicly available, however no URL is provided and a (somewhat cursory) web search does not reveal a download location (oddly the search does point to an earlier paper using the OADS that also suggests the dataset is available, but doesn't point to where).

Relatedly, the authors state "Original (non-blurred) stimuli will be made available upon request" - I much prefer, given modern standards, that the stimuli be made available along with the code on the github site.

Please proof the manuscript - there were various typos, odd word choices, etc. Examples:

p. 2, li 30: combining local sampling (inspired by pRF models) OF rich feature representations (from CNNs) - I think you mean with

p. 19, li 570: "Amsterdamapproved"

Reviewer #3: The authors build CNN-based linearized encoding models to predict ERP amplitudes elicited by large-field, high-resolution natural scenes. They compare four ways of handling spatial sampling of CNN feature maps: using the full feature map (“Full”), keeping only a tiny central crop (“Center”), removing that same central crop (“Periphery”), and applying a retinal ganglion cell sampling (GCS) transform that magnifies central regions while down weighting periphery (“GCS”). They report (i) improved encoding performance with GCS, (ii) distinct temporal profiles for center vs periphery information, (iii) an additional EEG experiment with explicit aperture stimulation to validate center/periphery temporal differences, and (iv) a “fully data-driven” random-sampling method to recover spatial tuning maps from EEG.

This is a well-executed study that does provide some new insights into the spatiotemporal use of visual information in complex natural scenes. There are several places where the clarity of the methodology can be improved. Further, although there are several fascinating takeaways from these analyses, the core contribution (coarse to fine processing with linearized encoding models) may not carry enough novelty for this journal.

-1- Novelty and contribution

The core pipeline (CNN features → PCA → linear regression → correlation with neural data) is standard for encoding work, and the core result (coarse to fine processing) has been established for some decades. That said, the authors' retina-inspired sampling of CNN activation maps is a nice contribution, especially when combined with the replication experiment (though with N=10, this seems a little tacked on at the end). I leave it up to the editor to determine what novelty threshold should be applied here.

-2- Several methods require more information to fully evaluate and/or need clarification

A. The spatial feature selection

It would be helpful to fully articulate that the spatial selection is done post hoc on CNN activation maps rather than re-deriving weights from pixel masking or some other procedure. It took me a couple of readings to get this straight.

B. Train/test split with different SNR

As I understand, the authors fit a low-SNR set (5 image repetitions) and then evaluate on the high-SNR set (10 repetitions). Can the authors please provide some rationale for this choice? Do the results still hold if they train on high-SNR and test on low? Or if the splits are equalized?

C. Random sampling of pixel-wise contribution map

Some critical implementation details are missing here. How were pixels selected (i.e., from what distribution?) How many pixels per iteration? Was this stratified by eccentricity? Further, it's not clear whether the scoring correlation was computed on the same data used to fit the regression weights. If so, I would like to see a cross-validated version of this analysis to truly test generalizability.

D. GCS parameterization

The authors used the default foveal size of 20 degrees. Given the paper's own suggestion that varying magnification could matter, it would be nice to vary this parameter to strengthen the claim.

-3- "Representative" results

A good chunk of the paper's figures use Iz and Pz as "representative electrodes." While I'm keenly sympathetic to the need to pare down such high-dimensional data, this also forces the reader to trust that these electrodes are in fact representative. A more standard approach might replace the single electrode traces with averaged ROIs that would represent posterior and central electrode clusters. Further, Iz is not a standard electrode in many montages, and in some participants, can be contaminated with artifacts from the neck muscles. Using ROI averages might even make the result even cleaner.

Smaller issues:

Figure 1 caption: GCS? Acronym should be specified. This is also true of Line 93 in the text.

Line 117: How were these timepoints chosen? I can see that at least 500 ms of post-stimulus information was recorded, so I'm curious why no later timepoints were considered. My initial thought was to avoid offset ERPs, given the RSVP presentation, but stimuli were on screen for 100 ms, so the last timepoint would also include an offset ERP.

Line 713: Since the first 100 PCs were used regardless of spatial selection process, some selections might have more retained information than others in these 100 PCs. Please report the variability retained across all manipulations.

Line 570: missing space

Line 633: missing space

Line 706: principal, not principle

**********

**Have the authors made all data and (if applicable) computational code underlying the findings in their manuscript fully available?**

The PLOS Data policy requires authors to make all data and code underlying the findings described in their manuscript fully available without restriction, with rare exception (please refer to the Data Availability Statement in the manuscript PDF file). The data and code should be provided as part of the manuscript or its supporting information, or deposited to a public repository. For example, in addition to summary statistics, the data points behind means, medians and variance measures should be available. If there are restrictions on publicly sharing data or code —e.g. participant privacy or use of data from a third party—those must be specified.

Reviewer #1: Yes

Reviewer #2: **No:**  I am unclear if stimuli fall into this bin. If so, then the stimuli are not readily available without requesting them.

Reviewer #3: Yes

**********

PLOS authors have the option to publish the peer review history of their article (what does this mean?). If published, this will include your full peer review and any attached files.

If you choose “no”, your identity will remain anonymous but your review may still be made public.

**Do you want your identity to be public for this peer review?**  For information about this choice, including consent withdrawal, please see our Privacy Policy.

Reviewer #1: **Yes:**  Fernanda L. Ribeiro

Reviewer #2: No

Reviewer #3: No

[NOTE: If reviewer comments were submitted as an attachment file, they will be attached to this email and accessible via the submission site. Please log into your account, locate the manuscript record, and check for the action link "View Attachments". If this link does not appear, there are no attachment files.]

**Figure resubmission:**

While revising your submission, we strongly recommend that you use PLOS’s NAAS tool (https://ngplosjournals.pagemajik.ai/artanalysis) to test your figure files. NAAS can convert your figure files to the TIFF file type and meet basic requirements (such as print size, resolution), or provide you with a report on issues that do not meet our requirements and that NAAS cannot fix.

After uploading your figures to PLOS’s NAAS tool - https://ngplosjournals.pagemajik.ai/artanalysis, NAAS will process the files provided and display the results in the "Uploaded Files" section of the page as the processing is complete. If the uploaded figures meet our requirements (or NAAS is able to fix the files to meet our requirements), the figure will be marked as "fixed" above. If NAAS is unable to fix the files, a red "failed" label will appear above. When NAAS has confirmed that the figure files meet our requirements, please download the file via the download option, and include these NAAS processed figure files when submitting your revised manuscript.

**Reproducibility:**

To enhance the reproducibility of your results, we recommend that authors of applicable studies deposit laboratory protocols in protocols.io, where a protocol can be assigned its own identifier (DOI) such that it can be cited independently in the future. Additionally, PLOS ONE offers an option to publish peer-reviewed clinical study protocols. Read more information on sharing protocols at https://plos.org/protocols?utm_medium=editorial-email&utm_source=authorletters&utm_campaign=protocols

# Author comment: Author response to Decision Letter 1

**DOI:** 10.1371/journal.pcbi.1014371.r002

23 Mar 2026

Attachment Submitted filename: Review_responses.pdf

# Peer review: Decision Letter 1

**DOI:** 10.1371/journal.pcbi.1014371.r003

**Authors:** Marieke Karlijn van Vugt, Christoph Strauch

27 May 2026

Dear Mr. Müller,

We are pleased to inform you that your manuscript 'Linking retinal sampling in neural encoding models to temporal profiles of visual processing in humans' has been provisionally accepted for publication in PLOS Computational Biology.

Before your manuscript can be formally accepted you will need to complete some formatting changes, which you will receive in a follow up email. A member of our team will be in touch with a set of requests.

Please note that your manuscript will not be scheduled for publication until you have made the required changes, so a swift response is appreciated.

IMPORTANT: The editorial review process is now complete. PLOS will only permit corrections to spelling, formatting or significant scientific errors from this point onwards. Requests for major changes, or any which affect the scientific understanding of your work, will cause delays to the publication date of your manuscript.

Should you, your institution's press office or the journal office choose to press release your paper, you will automatically be opted out of early publication. We ask that you notify us now if you or your institution is planning to press release the article. All press must be co-ordinated with PLOS.

Thank you again for supporting Open Access publishing; we are looking forward to publishing your work in PLOS Computational Biology.

Best regards,

Christoph Strauch

Academic Editor

PLOS Computational Biology

Marieke van Vugt

Section Editor

PLOS Computational Biology

***********************************************************

Apologies for the long turnover time. It's a pleasure to accept the manuscript in the current form - congratulations!

Reviewer's Responses to Questions

**Comments to the Authors:**

**Please note here if the review is uploaded as an attachment.**

Reviewer #1: I have no further suggestions. Congrats!

Reviewer #2: I appreciate the diligence of the authors in addressing my questions and those of the other reviewers.

**********

**Have the authors made all data and (if applicable) computational code underlying the findings in their manuscript fully available?**

The PLOS Data policy requires authors to make all data and code underlying the findings described in their manuscript fully available without restriction, with rare exception (please refer to the Data Availability Statement in the manuscript PDF file). The data and code should be provided as part of the manuscript or its supporting information, or deposited to a public repository. For example, in addition to summary statistics, the data points behind means, medians and variance measures should be available. If there are restrictions on publicly sharing data or code —e.g. participant privacy or use of data from a third party—those must be specified.

Reviewer #1: Yes

Reviewer #2: Yes

**********

PLOS authors have the option to publish the peer review history of their article (what does this mean?). If published, this will include your full peer review and any attached files.

If you choose “no”, your identity will remain anonymous but your review may still be made public.

**Do you want your identity to be public for this peer review?**  For information about this choice, including consent withdrawal, please see our Privacy Policy.

Reviewer #1: **Yes:**  Fernanda Lenita Ribeiro

Reviewer #2: No

# Editor report: Acceptance letter

**DOI:** 10.1371/journal.pcbi.1014371.r004

**Authors:** Marieke Karlijn van Vugt, Christoph Strauch

PCOMPBIOL-D-25-02062R1

Linking retinal sampling in neural encoding models to temporal profiles of visual processing in humans

Dear Dr Müller,

I am pleased to inform you that your manuscript has been formally accepted for publication in PLOS Computational Biology. Your manuscript is now with our production department and you will be notified of the publication date in due course.

The corresponding author will soon be receiving a typeset proof for review, to ensure errors have not been introduced during production. Please review the PDF proof of your manuscript carefully, as this is the last chance to correct any errors. Please note that major changes, or those which affect the scientific understanding of the work, will likely cause delays to the publication date of your manuscript.

Soon after your final files are uploaded, unless you have opted out, the early version of your manuscript will be published online. The date of the early version will be your article's publication date. The final article will be published to the same URL, and all versions of the paper will be accessible to readers.

For Research, Software, and Methods articles, you will receive an invoice from PLOS for your publication fee after your manuscript has reached the completed accept phase. If you receive an email requesting payment before acceptance or for any other service, this may be a phishing scheme. Learn how to identify phishing emails and protect your accounts at https://explore.plos.org/phishing.

Thank you again for supporting PLOS Computational Biology and open-access publishing. We are looking forward to publishing your work!

With kind regards,

Kannan R K Kuppusamy, B.TECH BIOTECHNOLOGY

PLOS Computational Biology | Carlyle House, Carlyle Road, Cambridge CB4 3DN | United Kingdom ploscompbiol@plos.org | Phone +44 (0) 1223-442824 | ploscompbiol.org | @PLOSCompBiol
